# Mammalian TatD DNase domain containing 1 (*TATDN1*) is a proteostasis‐responsive gene with roles in ventricular structure and neuromuscular function

**DOI:** 10.1111/febs.70077

**Published:** 2025-03-23

**Authors:** Gisel Barés, Aida Beà, Anna Sancho‐Balsells, Juan G Valero, David Aluja, Javier Inserte, Sandra García‐Carpi, Elisabet Miró‐Casas, Sara Borràs‐Pernas, Sara Hernández, Ana Martínez‐Val, Jesper V Olsen, Francesc Tebar, Xavier Cañas, Joan X. Comella, Patricia Pérez‐Galán, Marisol Ruiz‐Meana, Albert Giralt, Marta Llovera, Daniel Sanchis

**Affiliations:** ^1^ Cell Signaling and Apoptosis Group, Departament de Ciències Mèdiques Bàsiques Universitat de Lleida Spain; ^2^ IRBLleida Lleida Spain; ^3^ Departament de Biomedicina, Facultat de Medicina Institut de Neurociències, Universitat de Barcelona Spain; ^4^ Institut d'Investigacions Biomèdiques August Pi i Sunyer (IDIBAPS), Production and Validation Center of Advanced Therapies (Creatio), Faculty of Medicine and Health Science, Centro de Investigación Biomédica en Red sobre Enfermedades Neurodegenerativas (CIBERNED) Madrid Spain; ^5^ Department of Hematology‐Oncology Institut d'Investigacions Biomèdiques August Pi i Sunyer (IDIBAPS) Spain; ^6^ Centro de Investigación Biomédica en Red‐Oncología (CIBERONC) Barcelona Spain; ^7^ Cardiovascular Diseases Group Vall d'Hebron Institut de Recerca (VHIR), Vall d'Hebron Hospital Universitari and Universitat Autònoma de Barcelona Spain; ^8^ CIBER de Enfermedades Cardiovasculares (CIBER‐CV) Madrid Spain; ^9^ Experimental Neuromuscular pathology Group, Departament de Medicina Experimental Universitat de Lleida and IRBLleida Lleida Spain; ^10^ Novo Nordisk Foundation Center for Protein Research, Proteomics Program, Faculty of Health and Medical Sciences University of Copenhagen Denmark; ^11^ Departament de Biomedicina, Unitat de Biologia Cellular, Facultat de Medicina i Ciències de la Salut Universitat de Barcelona Spain; ^12^ Centre de Recerca Biomèdica CELLEX, Institut d'Investigacions Biomèdiques August Pi i Sunyer (IDIBAPS) Barcelona Spain; ^13^ Institut de Recerca Sant Joan de Deu Barcelona Madrid Spain; ^14^ Centro de Investigación Biomédica en Red Enfermedades Neurodegenerativas (CIBERNED), ISCIII Madrid Spain; ^15^ Present address: Universitat Pompeu Fabra Barcelona Spain; ^16^ Present address: Laboratory of Cardiovascular Proteomics, Centro Nacional de Investigaciones Cardiovasculares (CNIC) Madrid Spain

**Keywords:** cardiomyopathy, motor control, neurobehavior, TatD, Tatdn1, ventricle dilation

## Abstract

The characterization of highly conserved but poorly understood genes often reveals unexpected biological roles, advancing our understanding of disease mechanisms. One such gene is Mammalian TatD DNase domain containing 1 (*Tatdn1*), the mammalian homolog of bacterial Twin‐arginine translocation D (TatD), a protein proposed to have roles either in DNA degradation or protein quality control in unicellular organisms. Despite its association with different pathologies, including several cancer types and cardiovascular diseases, the role of TATDN1 in mammals remains unexplored. Here, we demonstrate that *Tatdn1* encodes a cytoplasmic protein that does not participate in DNA degradation but is upregulated in cells under proteostasis stress. *Tatdn1*‐deficient mice exhibit dysregulated expression of genes involved in membrane and extracellular protein biology, along with mild dilated cardiomyopathy and impaired motor coordination. These findings identify TATDN1 as a key player in cytosolic processes linked to protein homeostasis, with significant physiological implications for cardiac and neurological function.

## Graphical abstract

Twin‐arginine translocation D (TatD) homologs are found across all kingdoms, with debated roles in DNA degradation and protein quality control in unicellular organisms. We characterize TATDN1, the mammalian TatD orthologue, as a proteostatic stress‐responsive cytosolic protein dispensable for DNA degradation. *Tatdn1*‐knockout mice reveal TATDN1's relevant functions in the heart and nervous system.

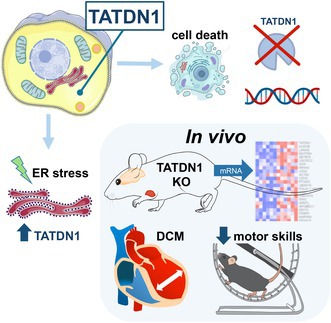

**Abbreviations**
CHEF: contour‐clamped homogeneous electric field electrophoresis
DCM: dilated cardiomyopathy
DMSO: dimethyl sulfoxide
dsDNA: double‐strand DNA
EF: ejection fraction
ER: endoplasmic reticulum
FS: fractional shortening
GSEA: Gene Set Enrichment Analysis
GWAS: gene‐wide association study
IFN: α interferon alpha
IVS: interventricular septum thickness
LVEDD: left ventricular internal diameter
LVEDV: left ventricular internal volume
LVESD: end‐systolic left ventricular internal diameter
LVPW: left ventricle posterior wall thickness
NET: neutrophil extracellular trap
NORT: novel object recognition test
ORF: open reading frame
shRNA: small hairpin RNA
ssDNA: single‐strand DNA
STS: staurosporine
TatD: Twin‐arginine translocation D
TATDN1: TatD DNase domain containing 1
T‐SAT: T‐spontaneous alteration task

## Introduction

Nucleases are central to the DNA degradation processes that occur during cell death, a phenomenon important for tissue homeostasis and response to injury. In exploring the roles of these enzymes in mammalian heart and brain tissues, we focused on the mitochondrial nuclease ENDOG and TATDN1. These nucleases were selected based on their reported roles in unicellular organisms and nematodes, where they mediate DNA degradation and developmental cell death [1, 2, 3]. While ENDOG has been extensively characterized, particularly in the myocardium [4, 5, 6, 7] and neurons [8], the physiological roles of TATDN1 remain largely unknown, despite its association with human pathologies, including several cancer types and dilated cardiomyopathy [9, 10].

TatD is a highly conserved protein found across all kingdoms. In bacteria, TatD is part of the *tatABCD* operon, which mediates the translocation of folded proteins with twin‐arginine motifs to the periplasm [2, 11, 12, 13]. Unlike its membrane‐bound counterparts [14] (TatA, B, and C), bacterial TatD is a soluble, cytoplasmic protein with proposed roles in Mg^2+^‐dependent DNA degradation, DNA repair, and protein quality control [2, 15, 16]. Notably, the Tat protein transport system is absent in eukaryotes [17], yet TatD homologs are conserved [18]. Functional studies of bacterial TatD and its eukaryotic homologs have primarily relied on overexpression systems and *in vitro* conditions [2, 16, 18, 19, 20]. Despite hypothesized nuclear localization, their biological roles in DNA degradation have not been demonstrated *in vivo*, leaving their physiological impact speculative. In unicellular eukaryotes, TatD‐like nucleases are implicated in DNA degradation [18, 21, 22] and processes like neutrophil extracellular trap (NET) degradation by *Plasmodium falciparum* [20]. However, these findings are limited to studies with recombinant proteins, and the relevance of TatD proteins to DNA degradation in living cells is unproven.

In mammals, TATDN1 has been identified as the homolog of the bacterial TatD and the apoptotic nucleases characterized in yeast (*YBL055C*) and *C. elegans* (*crn‐2*) [3, 18]. Eukaryotic TatD‐like proteins are proposed to play roles in apoptosis and embryonic development, yet functional studies of mammalian TATDN1 have been limited to *in vitro* recombinant protein assays [19], leaving its *in vivo* roles unexplored.

Given its evolutionary conservation and proposed apoptotic roles, we hypothesized that TATDN1 may have critical functions in mammalian cell death and development. In this study, we investigated TATDN1 expression and function in mammalian cells. We found that TATDN1 is a ubiquitously expressed cytosolic protein, dispensable for DNA degradation during cell death, yet responsive to proteostatic stress. Loss of TATDN1 in mice led to dysregulation of genes encoding membrane‐bound and extracellular proteins, mild dilated cardiomyopathy, and impaired motor coordination. These findings shed light on the unexpected physiological roles of TATDN1 and its potential relevance to human diseases.

## Results

### TATDN1 is the mammalian ortholog of bacterial TatD and is a ubiquitously expressed cytoplasmic protein unrelated to DNA degradation

The information available in several genome databases of model organisms (Fig. S1), as well as our alignment analysis (Fig. S2), pointed to *Tatdn1* as the mammalian ortholog of the bacterial *TatD* gene. Human TATDN1 is a 297 aa long, 33.6 kDa polypeptide (https://www.ncbi.nlm.nih.gov/protein/NP_114415.1) predicted to have a globular structure, based on X‐ray diffraction analysis data (https://alphafold.ebi.ac.uk/entry/Q6P1N9). TATDN1 contains a conserved ion‐dependent hydrolase domain (https://www.ncbi.nlm.nih.gov/Structure/cdd/cddsrv.cgi) and has a presumed 3′‐5′‐exodeoxyribonuclease domain (https://www.uniprot.org/uniprot/Q6P1N9#function). The Human Protein Atlas (HPA) suggests that TATDN1 has nucleoplasmic localization in U‐251 MG glioblastoma astrocytoma, based on immunofluorescence detection with an anti‐TATDN1 antibody (http://www.proteinatlas.org/ENSG00000147687‐TATDN1/cell). In contrast, the TatD orthologue from yeast, YBL055C, tagged at the carboxy terminal end with green fluorescent protein was localized to the cytoplasm [23]. Sequence alignment at the protein level showed high conservation among 15 selected species ranging from bacteria (*E. coli*) to humans (Fig. S2). Two motifs of ≥10 aa are highly conserved among the 15 sequences analyzed. These motifs contain putative metal‐binding residues, as described for bacterial TatD [16], and the nuclease domain as identified in the TATDN1 UniProt entry (https://www.uniprot.org/uniprotkb/P27859/entry) (Fig. S2).

**Fig. 1:**
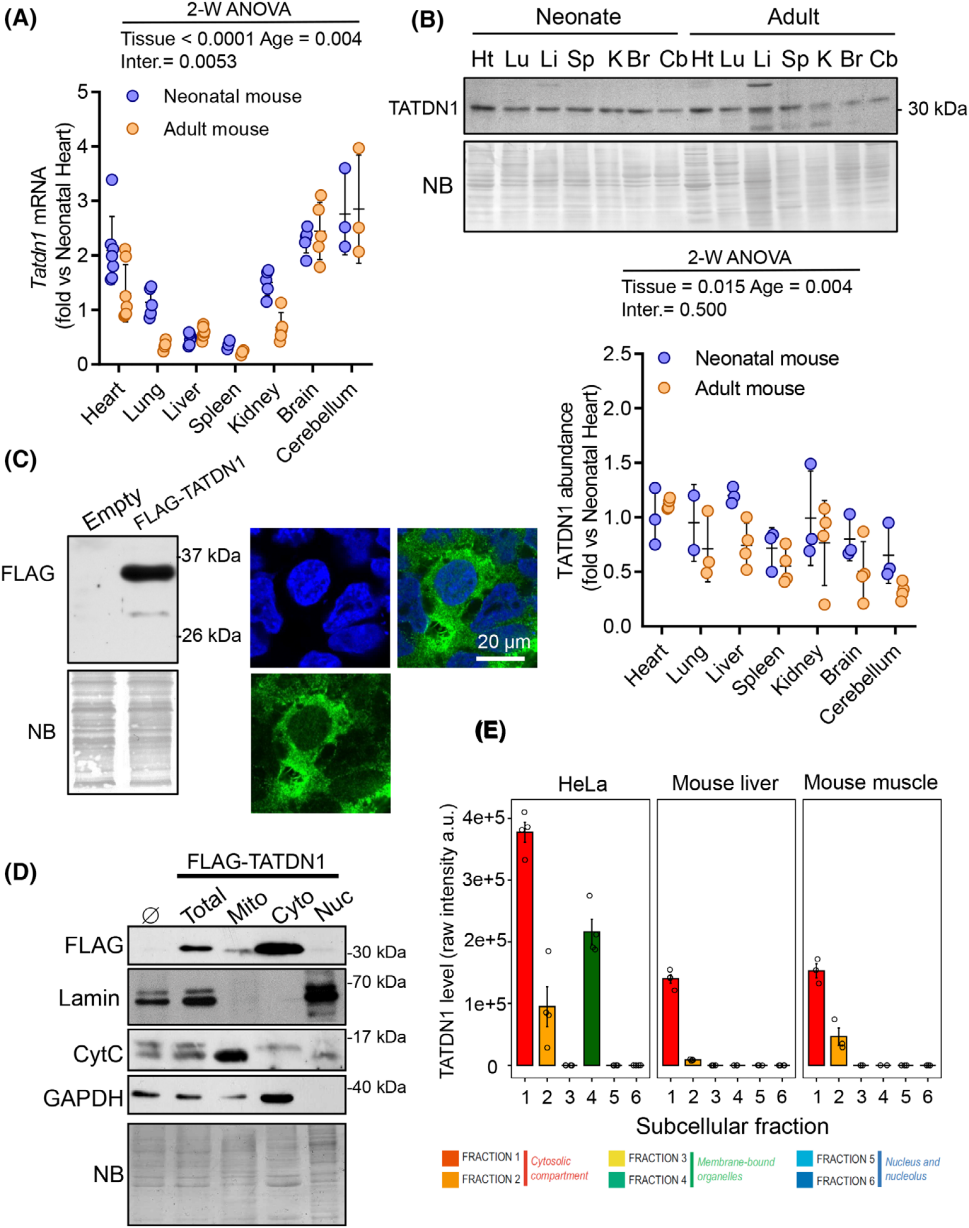
*Tatdn1* gene is ubiquitously expressed and FLAG‐TATDN1 has a cytoplasmic localization. (A) RT‐qPCR of Tatdn1 in 3‐ to 5‐day‐old neonatal (blue) and 4–5‐month‐old adult (orange) mouse tissues: Br, brain; Cb, cerebellum; Ht, heart; K, kidney; Lu, lung; Li, liver; Sp, spleen. Each individual value from 3 to 7 replicates is shown plus mean ± SD. (B) Representative TATDN1 western blot from total protein extracts obtained from mice in (A). NB, Naphthol blue stained membrane for loading control. Densitometric quantifications of TATDN1 band for each tissue referred to neonatal heart signal are depicted in the graph below. Each value from triplicates is shown plus mean ± SD (C) Representative western blot and confocal immunofluorescence images of overexpressed FLAG‐TATDN1 in HEK293 cells. Green: FLAG‐TATDN1, blue: Hoechst staining (nuclei) and merged images are shown. (D) Western blot of protein extracts and subcellular fractions of HEK293 cells transfected with empty vector (ø) or FLAG‐TATDN1 vector. Cyto, cytoplasm; Mito, mitochondria; Nuc, nucleus. Markers: CytC, cytochrome c for mitochondria; GAPDH for cytoplasm; Lamin A + C for nuclei. NB, Naphthol blue membrane staining. For A and B, two‐way ANOVA followed by Sidak's test was run. Inter. = Interaction. (E) Bar plots: ± SEM showing TATDN1 levels as raw intensity obtained from mass spectrometry analysis of six subcellular fractions in HeLa cells (*n* = 4), mouse liver (*n* = 4) and mouse muscle (*n* = 3) from Martinez‐Val *et al*. [24].

Experimentally, we analyzed *Tatdn1* expression at the transcript level in several neonatal and adult mouse tissues. Quantitative PCR after reverse transcription of total RNA showed that the *Tatdn1* transcript was expressed in the heart and nervous system but was also abundant in other organs (Fig. 1A). TATDN1 protein was detected at levels not directly related to the transcript abundance in all tissues analyzed (Fig. 1B), suggesting tissue‐specific regulation of translation and/or protein turnover. Moreover, in some organs, such as the liver, brain, and cerebellum, a tendency to decrease in adulthood was observed. Analysis of TATDN1 abundance in primary cultures of neonatal rat cardiomyocytes, cardiac fibroblasts, cortical neurons, and glia showed no differences related to cell type (Fig. S3a). Immunofluorescence detection of FLAG‐tagged mouse TATDN1 in transfected human HEK293 cells showed a cytoplasmic staining (Fig. 1C). Due to the unexpected localization compared to the HPA annotation, we performed FLAG‐TATDN1 detection in subcellular fractions of FLAG‐TATDN1‐transfected HEK293 cells, which confirmed the cytoplasmic localization of overexpressed TATDN1 (Fig. 1D). An additional confirmation of these results was achieved by exploring publicly available spatial proteomics data by Martinez‐Val *et al*. [24]. Human and mouse endogenous TATDN1 were found in cytosolic fractions of HeLa cells and mouse liver and skeletal muscle, whereas it was absent in fractions corresponding to nuclei [24] (Fig. 1E; Fig. S3b). Moreover, an independent proteome‐wide mapping study of protein localization performed on human cancer cell lines corroborated the cytosolic localization of human TATDN1 (Fig. S4) [25].

**Fig. 2:**
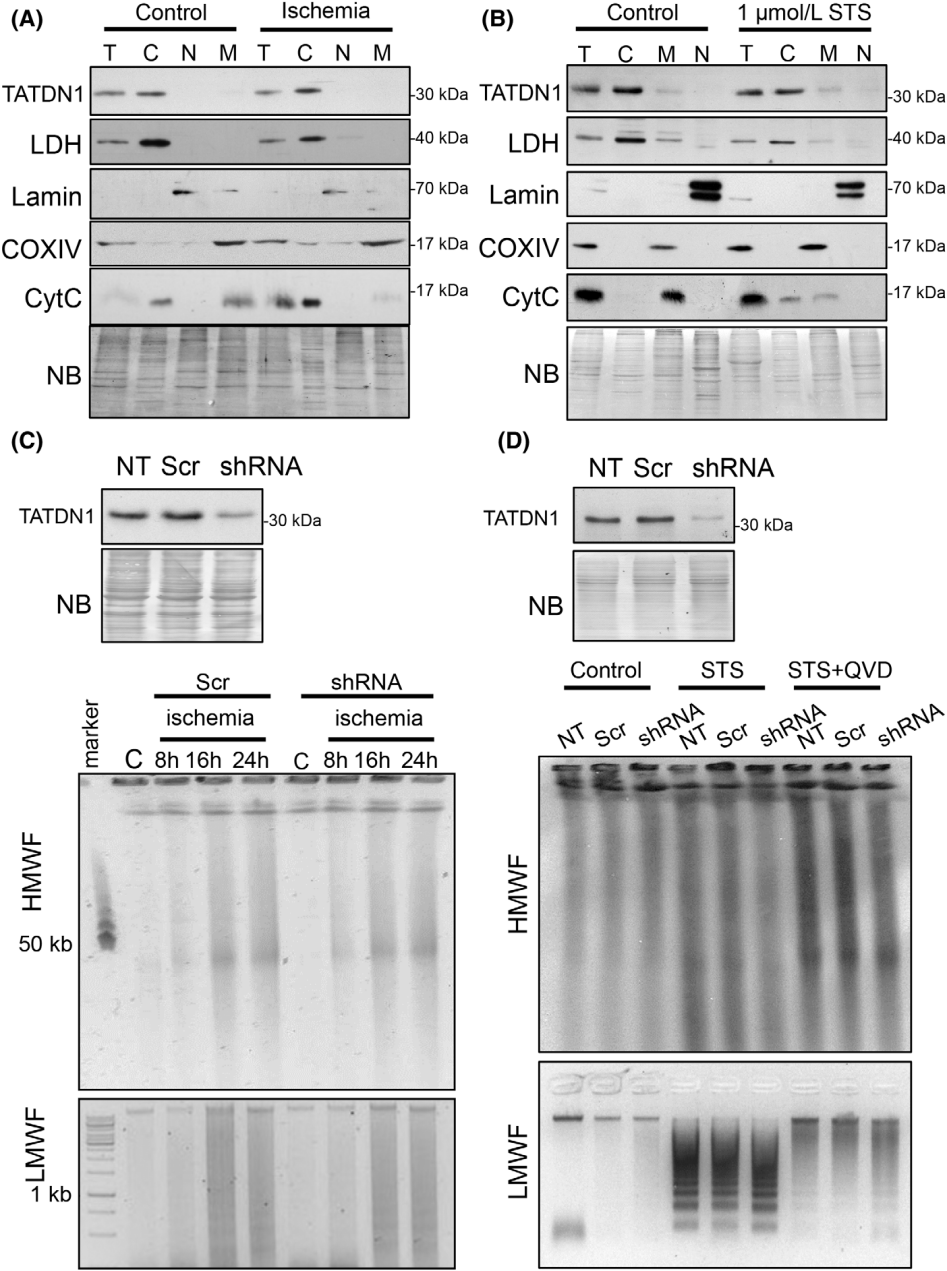
TATDN1 cytoplasmic localization is not altered during cell death and its expression is not required for caspase‐independent and caspase‐dependent DNA degradation. Representative western blot showing endogenous TATDN1 expression in total (T), cytosolic (C), nuclear (N) and mitochondrial (M) subcellular fractions of (A) primary neonatal rat ventricular cardiomyocytes cultured in normal conditions or submitted to 12 h ischemia; or (B) primary neonatal dermal fibroblasts treated with 1 μmol·L^−1^ staurosporine (STS) for 14 h. *N* = 3 independent experiments. Subcellular fraction purity was assessed by blotting fragments of the membranes with antibodies against lactate dehydrogenase (LDH; cytoplasm), Lamin A + C (Lamin, nucleus), cytochrome oxidase‐IV (COXIV, mitochondrial membrane), cytochrome c (CytC, mitochondria but released during cell death). NB, Naphthol blue total protein staining of the membrane. (C) TATDN1 western blot for the assessment of the efficiency of lentiviral‐driven *Tatdn1* gene silencing in primary rat neonatal cardiomyocytes (upper panel) and DNA agarose gel electrophoresis images of total DNA extracts from cardiomyocytes that were cultured in control conditions or in experimental ischemia for 8, 16, and 24 h. (D) TATDN1 western blot for the assessment of the efficiency of lentiviral‐driven *Tatdn1* gene silencing in Rat2 cells (upper panel) and DNA agarose gel electrophoresis images of total DNA extracts from control fibroblasts and cultures treated with 1 μmol·L^−1^ staurosporine (STS) and STS + 5 μmol·L^−1^ Q‐VD‐OPh (QVD; pan‐caspase inhibitor) for 8 h. NT, not transduced; Scr, scrambled‐transduced; shRNA, *Tatdn1*‐specific silencing. HMWF, DNA high molecular weight fragmentation; LMWF, DNA low molecular weight fragmentation. Representative images are shown from three independent experiments. Marker: 50 kb DNA molecular weight marker (for CHEF) and 1 kb DNA marker (for conventional electrophoresis). NB, Naphthol blue staining.

To complement overexpression results and proteomic data, we detected endogenous TATDN1 in several subcellular fractions of primary neonatal rat cardiomyocytes and dermal fibroblasts with a specific commercial antibody. Native TATDN1 was exclusively detected in the cytoplasm of both cell types (Fig. 2A,B). Because the mitochondrial nuclease ENDOG translocates to the nucleus during cell death [1], we checked whether TATDN1 could also change its location during caspase‐dependent and independent cell death. However, we did not detect TATDN1 translocation to the nucleus during experimental ischemia in cultured cardiomyocytes, a model of caspase‐independent death [4], or in Rat‐2 cells treated with the multikinase inhibitor staurosporine (STS), which induces caspase‐dependent cell death in fibroblasts [4] (Fig. 2A,B). We also investigated the potential role of TATDN1 in caspase‐dependent and independent DNA degradation. Cardiomyocytes expressing low TATDN1 levels due to lentiviral‐driven *Tatdn1*‐specific shRNA‐induced silencing (Fig. 2C, western blot) showed identical ischemia‐induced high molecular and low molecular weight DNA degradation, which is caspase‐independent [4], compared to the scrambled‐transduced cardiomyocytes (Fig. 2C). *Tatdn1* expression silencing in Rat‐2 fibroblasts (Fig. 2D, western blot) did not modify high molecular weight DNA degradation and DNA laddering, after treatment with STS (Fig. 2D). In contrast, the presence of Q‐VD‐OPh, a caspase inhibitor, reduced DNA low molecular weight degradation, showing that this event was dependent on caspase activation. Together, our results show that *Tatdn1* is expressed in neonatal and adult mouse tissues and that TATDN1 has a cytoplasmic location, which is neither altered in situations of caspase‐dependent nor caspase‐independent cell death. Furthermore, TATDN1 is not required for caspase‐dependent and independent genomic DNA degradation in rat cells.

### TATDN1 is not required for mouse embryonic and postnatal development and cell proliferation

**Fig. 3:**
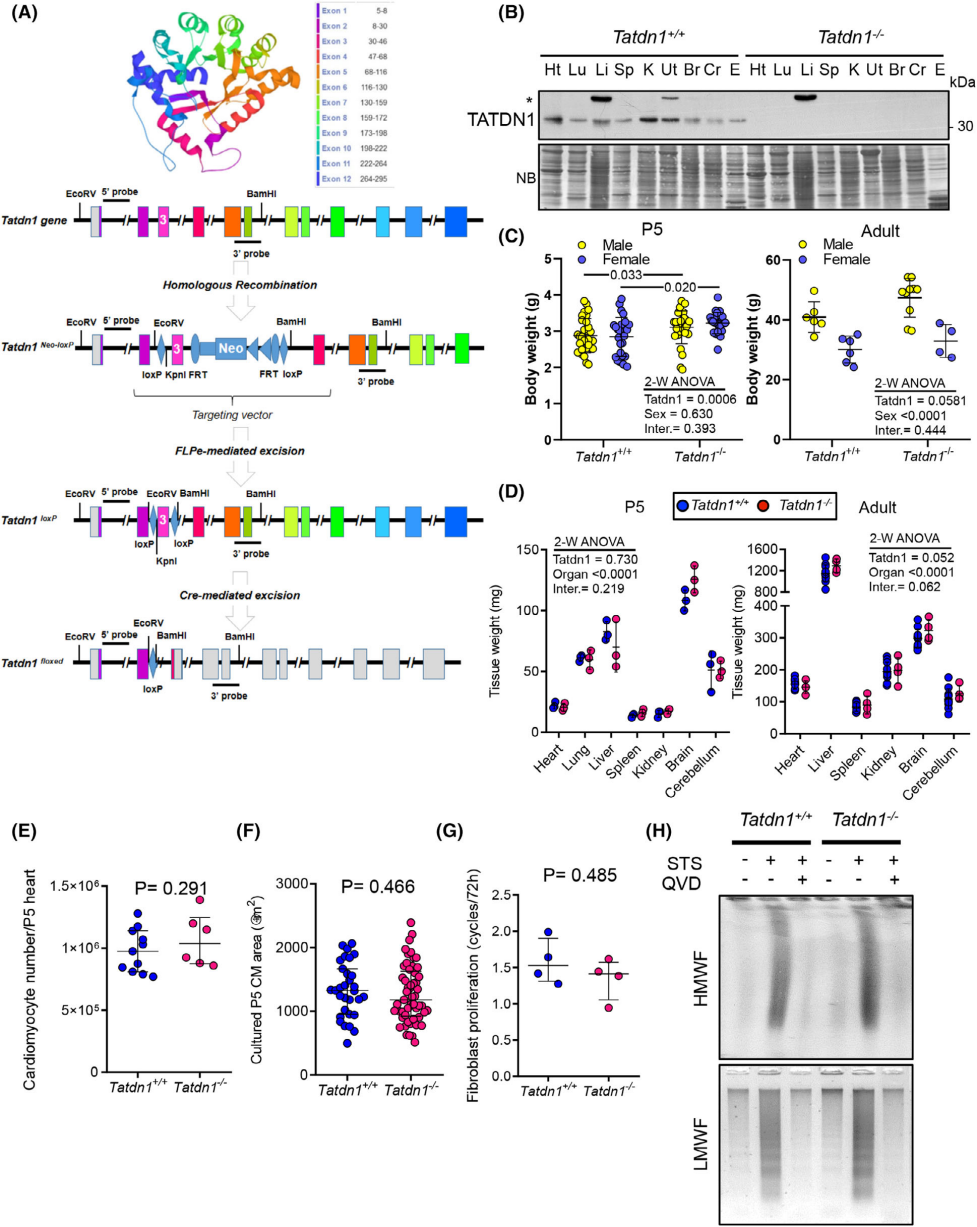
TATDN1 is dispensable for embryo and postnatal development, cell proliferation, and cell death‐associated DNA degradation in mice. (A) Strategy to generate the conditional *Tatdn1* allele. Three‐dimensional model of human TATDN1 (UniProt Q6P1N9) associating the corresponding coding exons to their location in the polypeptide chain, *Tatdn1* exonic structure indicating the Southern blot probes, and targeted allele showing location of the fragment integrated from the targeting vector. After homologous recombination, the Neomycin resistance cassette is located downstream exon 3, flanked by *loxP* and *FRT* sites. The Neomycin cassette was removed by crossing *Tatdn1* floxed homozygous mice with *ROSA‐FLPe* mice. Cre‐mediated excision resulted in one *loxP* site in the place of exon 3. (B) TATDN1 protein expression in adult *Tatdn1* ^*+/+*^ and *Tatdn1* ^*−/−*^ mouse tissues by western blot. Br, brain; Cb, cerebellum; E, eye; Ht, heart; K, kidney; Lu, lung; Li, liver; Sp, spleen; Ut, uterus. *Denotes unspecific band. NB, Naphthol blue protein staining of the membrane (representative image from 3 independent experiments). (C) Body weight of P5 pups (26–30 per gender and genotype), and P90 mice (4–10 per gender and genotype). (D) Organ weight of P5 (3 males/genotype) and P90 males (10 *Tatdn1* ^*+/+*^, 4*Tatdn1* ^*−/−*^). Individual values are plotted with mean ± SD (C, D). (E) cardiomyocyte number/heart counted as described in the Methods section, from 11 *Tatdn1* ^*+/+*^ and 6 *Tatdn1* ^*−/−*^ P5 pups. (F) Area of P5 cultured cardiomyocytes calculated as described in the Methods section. Dots correspond to individual cardiomyocyte areas counted in several microscopic fields of two independent neonatal cardiomyocyte cultures per genotype (>50 cells/genotype are plotted). (G) Number of cell cycles completed in 72 h of primary dermal fibroblasts from *Tatdn1* ^*+/+*^ and *Tatdn1* ^*−/−*^ neonatal mice. An equal number of primary dermal fibroblasts were simultaneously seeded, and 72 h later, cells were detached and counted. *N* = 4 independent experiments. Median ± interquartile range is also indicated (E–G). (H) Analysis of DNA degradation of primary dermal fibroblasts from *Tatdn1* ^*+/+*^ and *Tatdn1* ^*−/−*^ neonatal mice. Fibroblasts were cultured and treated or not with 1 μmol·L^−1^ staurosporine (STS) for 8 h in the presence or absence of 5 μmol·L^−1^ of the pan‐caspase inhibitor Q‐VD‐OPh (QVD). DNA was extracted and electrophoresed and stained as described in the Methods section. HMWF, DNA high molecular weight fragmentation; LMWF, DNA low molecular weight fragmentation. *N* = 3 independent times with similar results. Plots show individual experimental values, medians, and interquartile ranges. Two‐way ANOVA followed by Sidak's test (C, D) and Mann–Whitney U‐test (E–G) were run. Inter., Interaction. Statistical differences are indicated in each graph.

*TatD* gene orthologs are present in a broad number of species and have been reported to affect DNA integrity [16, 18, 19, 20, 21, 22] protein transport^6^ and embryo development [3]. Therefore, we decided to analyze the effects of *Tatdn1* deficiency *in vivo* in mice, as a mammalian model. The knockout strategy design and execution was performed by the Australian Regenerative Medicine Institute (ARMI) at MONASH University (Fig. 3A; Fig. S5). *LoxP* sites were introduced flanking mouse *Tatdn1* exon 3 by electroporation of ES cells with a plasmid containing the floxed *Tatdn1* exon 3 and a neomycin resistance cassette flanked by FRT sites, to induce an ORF shift after Cre‐dependent fragment deletion, generating a stop codon in exon 4 (Fig. 3A; Fig. S5a–f). *Tatdn1* floxed chimera were crossed with C57BL/6 females and bred to homozygosis (Fig. 3A; Fig. S5f). Then, *Tatdn1* floxed homozygous mice were crossed with ROSA‐FLPe mice to delete the neomycin cassette used for transgenic ES cell selection (Fig. 3A; Fig. S5g) and homozygous descendants were crossed with CAG‐*Cre* mice to induce *Tatdn1* exon 3 deletion (Fig. S5h). CAG‐Cre transgene was eliminated by crossing *Tatdn1* ^*+/+*^ *Cre* ^*−/−*^ and *Tatdn1* ^*−/−*^ *Cre* ^*+/−*^ mice, and *Tatdn1* ^*+/−*^ *Cre* ^*−/−*^ descendants were intercrossed to obtain homozygous wild‐type and knockout mice (Fig. S5i). Analysis of TATDN1 protein abundance in adult *Tatdn1* ^*+/+*^ and *Tatdn1* ^*−/−*^ mice showed its absence in all knockout tissues analyzed (Fig. 3B) and served to validate the specificity of the antibody used to demonstrate the cytoplasmic location of TATDN1 in rat cardiomyocytes and fibroblasts. No changes were observed due to *Tatdn1* gene deletion in breeding efficiency (7.2 ± 2.0 and 8.1 ± 2.7 pups·L^−1^ for *Tatdn1* ^*+/+*^ and *Tatdn1* ^*−/−*^, respectively; mean ± SD *n* = 11; *t*‐test *P* = 0.586). Body weight was slightly higher in *Tatdn1* ^*−/−*^ neonates at 5 days *postpartum* but was not significantly different in adults (Fig. 3C). Lack of genotype‐associated body weight differences was maintained after correcting by tibia length (Fig. S6). No differences in organ weight due to genotype were observed (Fig. 3D). Neonates of both genotypes had similar cardiomyocyte number (Fig. 3E) and area (Fig. 3F). *Tatdn1* ^*+/+*^ and *Tatdn1* ^*−/−*^ skin fibroblasts had similar proliferation rates (Fig. 3G). Furthermore, treatment of *Tatdn1* ^*+/+*^ and *Tatdn1* ^*−/−*^ primary dermal fibroblasts with STS had the same effects on DNA degradation (Fig. 3H). Together, these results suggest that TATDN1 is dispensable for mouse embryo and postnatal development, cell proliferation, and DNA degradation associated with two different types of cell death (caspase‐dependent and independent).

**Fig. 4:**
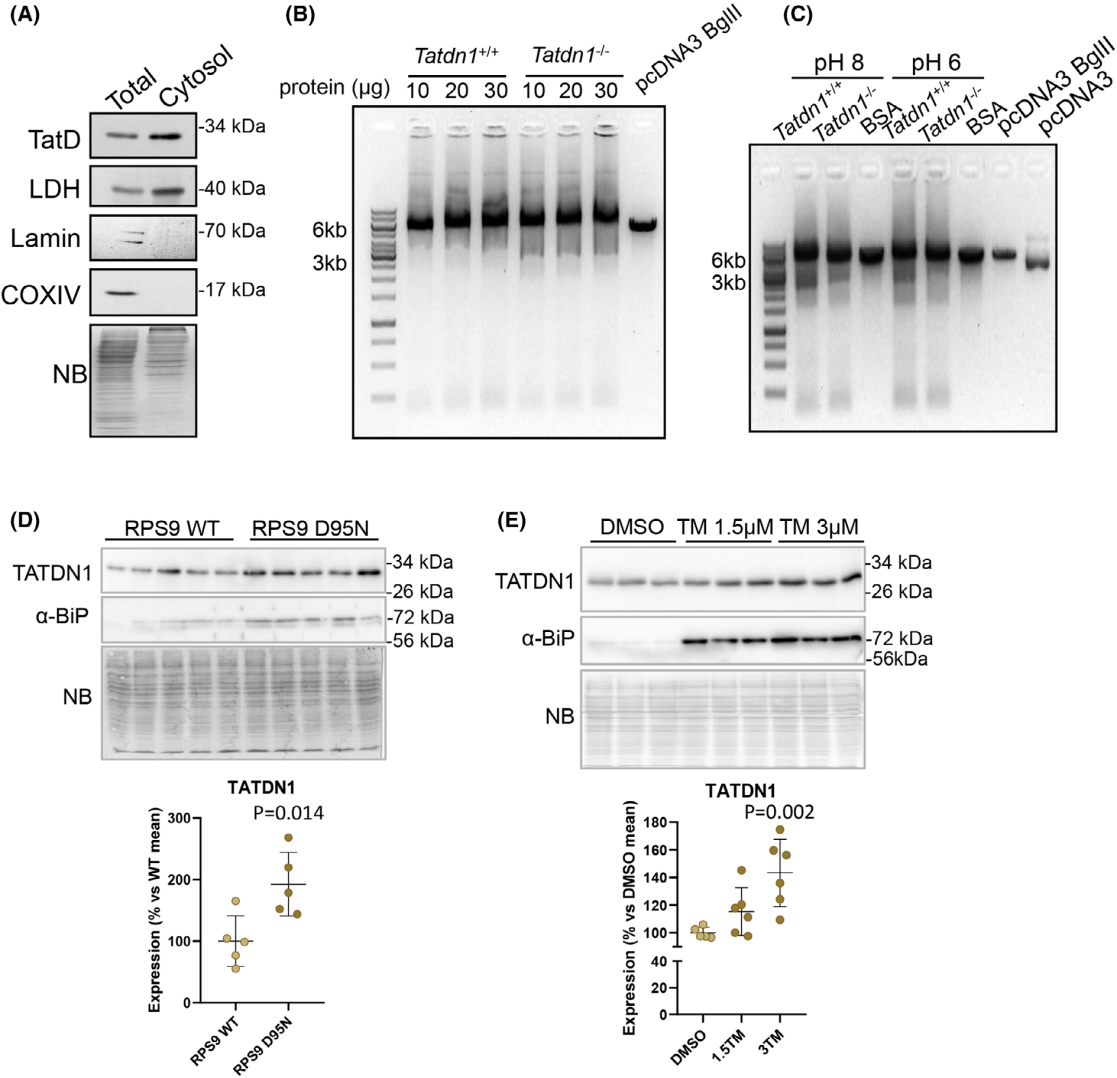
DNA degradation *in vitro* assays do not support DNase activity of endogenous TATDN1. (A) Assessment of TATDN1 enrichment in cytosolic extracts (cytosol) compared to total cell lysates (Total) of rat primary dermal fibroblasts. Potential contamination of cytosolic fractions with other subcellular compartments was assessed by blotting different fragments of the same membranes with antibodies against lactate dehydrogenase (LDH; cytoplasm), Lamin A + C (Lamin, nucleus), cytochrome oxidase‐IV (COXIV, mitochondrial membrane). NB, Naphthol blue membrane protein staining. (A) Representative sample from 3 independent experiments used in DNase assays is shown. (B) 1 μg of BglII‐linearized pcDNA3.1 plasmid (5.4 kb) was incubated with different amounts of cytosolic extracts from *Tatdn1* ^*+/+*^ and *Tatdn1* ^*−/−*^ dermal fibroblast cultures (10, 20, 30 μg of protein) for 2 h at 37 °C and pH 8 in the conditions described in Methods. (C) Cytosolic extracts (30 μg protein) from *Tatdn1* ^*+/+*^ and *Tatdn1* ^*−/−*^ dermal fibroblast cultures or 30 μg of BSA were incubated with 1 μg of BglII‐linearized pcDNA3.1 plasmid for 2 h at 37 °C and pH 8 or 6. At the end of the incubation time, the samples and aliquots of BglII‐digested and nondigested plasmid were electrophoresed in 1% agarose gels stained with SYBR Safe, and images were captured under UV illumination. A marker of DNA molecular weight was loaded at the left of each gel (6 and 3 kb bands are identified in the figure). Each assay was repeated three times with equal results. (D) TATDN1 expression in total protein extracts of HEK293 transfected with the wild‐type Rps9 or the Rps9 D95N mutant form inducing mistranslation (*N* = 5 clones each for RPS9 WT and D95N; mean ± SD), unpaired *t*‐test was used to determine statistically significant differences. (E) TATDN1 expression in total protein extracts of HEK293 cells treated for 24 h with tunicamycin at two different concentrations to induce UPR (*N* = 5 DMSO; *N* = 6/tunicamycin 1.5 and 3 μmol·L^−1^; mean ± SD). One‐way ANOVA followed by Dunnett's test was performed to assess statistically significant differences. Analysis of the endoplasmic reticulum chaperone α‐BiP was used to evidence activation of the unfolded protein response.

All the published experiments supporting DNase activity of bacterial TatD and its orthologs were performed using overexpression setups [15, 16, 18, 19, 20, 21] and, whereas Mg^2+^ was shown to be required for bacterial and yeast protein DNase activity [2, 16, 18], plasmodium TatD ortholog nuclease activity was inhibited by this cation [20] (summarized in Supplemental File 1_Fig. 3). To gain further insight into the possible DNase function of mammalian TATDN1, we assessed the capability of cytoplasm‐enriched fractions of *Tatdn1* ^*+/+*^ and *Tatdn1* ^*−/−*^ primary dermal fibroblasts to degrade a linearized plasmid *in vitro*. Our assay was based on the procedure published by Gannavaran *et al*. [21]. Despite the enriched abundance of TATDN1 in the wild‐type cytosolic extracts (Fig. 4A), different amounts of cytosolic extracts from *Tatdn1* ^*+/+*^ and *Tatdn1* ^*−/−*^ cells did not degrade several amounts up to 1 μg of BglII‐linearized pcDNA3 plasmid (Fig. 4B). Some pcDNA3 degradation was induced by both cytosolic protein extracts, either at pH 8 or 6 as compared to BSA addition, but at a similar extent by *Tatdn1* ^*+/+*^ and *Tatdn1* ^*−/−*^ cytoplasms (Fig. 4C). Together, under the experimental conditions tested, these results do not support deoxyribonuclease activity of TATDN1 present in the cytosolic extracts of mouse fibroblasts against dsDNA.

The above results demonstrate that TATDN1 localizes to the cytosol under both normal and apoptotic conditions, challenging its proposed role in DNA degradation in mammalian cells. Alternatively, it could be involved in protein biology as reported in bacteria [15]. In HEK293 cells, TATDN1 abundance was elevated in five independent clones carrying the RPS9 D95N ribosomal ambiguity mutation, which causes genome‐wide error‐prone translation, compared to controls [26] (Fig. 4D). Similarly, TATDN1 expression was dose‐dependently increased in HEK293 cells by Tunicamycin, an inducer of proteostatic stress [27] (Fig. 4E). Collectively, these results suggest that TATDN1 is a proteostatic stress‐responsive gene in mammalian cells.

### Comparative gene expression analysis suggests that mammalian TATDN1 functions are related to cellular membranes and vesicle trafficking but not to DNA degradation

**Fig. 5:**
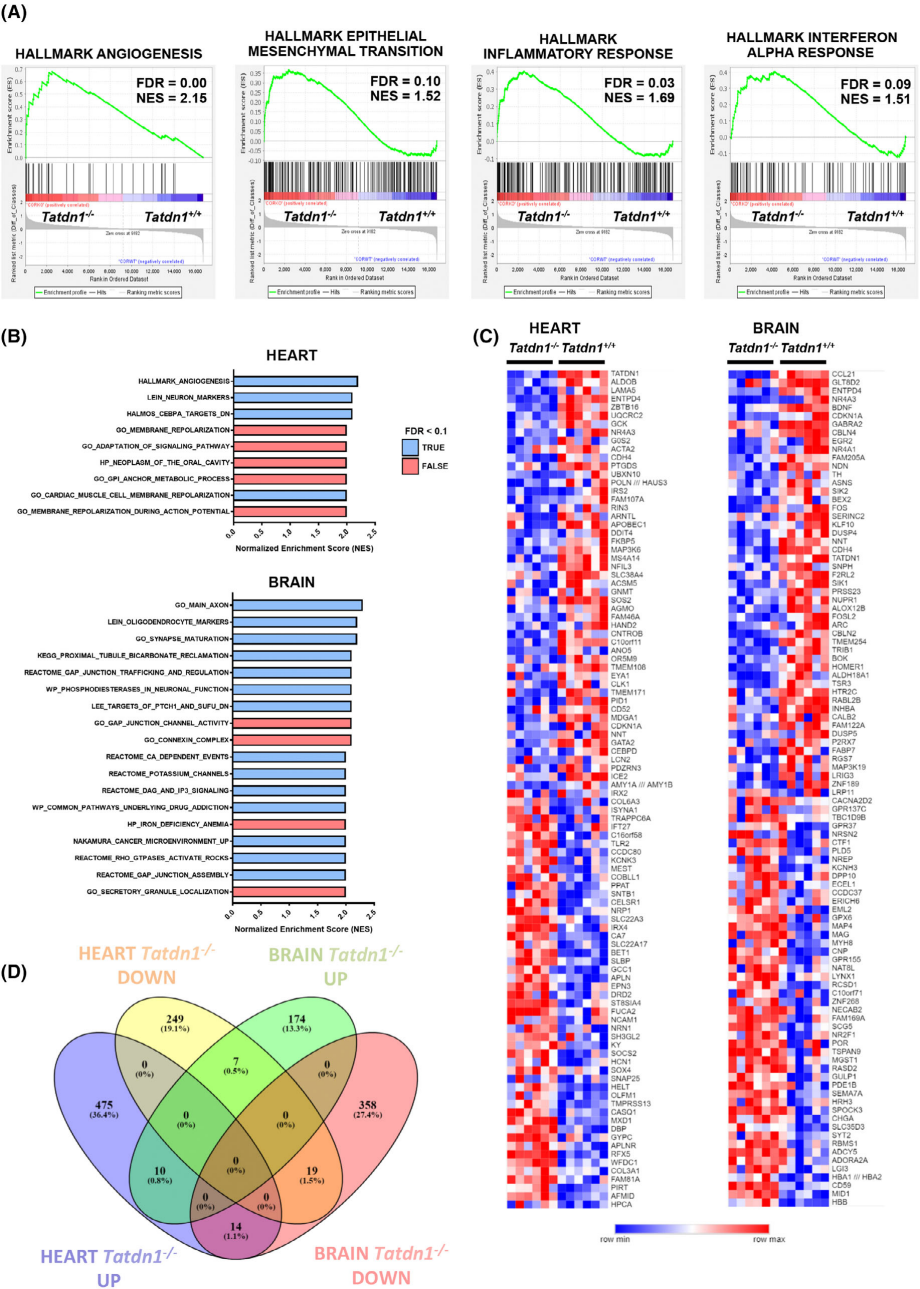
Analysis of the gene expression changes induced by Tatdn1 deletion in the heart ventricle and brain cortex of adult mice. An expression microarray was performed comparing *Tatdn1* ^*+/+*^ (WT) and *Tatdn1* ^*−/−*^ (KO) mice as described in the Methods section (*n* = 6/genotype). (A) Data mining analysis was done with GSEA software (FDR < 0.05, NES > 1.5). Representative enrichment plots of angiogenesis, epithelial mesenchymal transition, inflammatory response, and interferon alpha response gene sets are shown. FDR, false discovery rate; NES, normalized enrichment score. (B) Gene sets overrepresented in the heart and brain cortex. GSEA was used to test for significant enrichment of defined gene signatures. FDR, false discovery rate; NES, normalized enriched score. Threshold FDR < 0.1 and NES ≥ 2.0. Hallmark, C2, and C5 gene sets were obtained from the Molecular Signature Database (v2.5). (C) Heat map showing the top 100 genes from the ranked list of GSEA differentially expressed (upregulated and downregulated in heart and brain cortex and ordered by fold change). The Wilcoxon test was used to determine statistical significance. (D) Venn diagram showing the common upregulated and downregulated genes (FC >1.5, FDR <0.05) in the heart and brain cortex. Transcriptome analysis was carried out with Transcriptome Analysis Console software (Applied Biosystems, Thermo Fisher Scientific). Gene names and human orthologues of common genes are specified in Data S1.

To determine the molecular changes induced by *Tatdn1* gene disruption, we performed a microarray‐based gene expression analysis comparing adult *Tatdn1* ^*+/+*^ and *Tatdn1* ^*−/−*^ mice's cardiac ventricle and brain cortex. Due to the ubiquitous expression of *Tatdn1* (Figs 1B, 3B), we decided to initially analyze changes in the heart and the brain, two organs in which our group has previously found roles of apoptotic regulators in cell death and in organ morphology and function [6, 7, 28, 29, 30]. The Gene Set Enrichment Analysis (GSEA) method identified the upregulation of genes involved in angiogenesis, epithelial mesenchymal transition, inflammation, and response to IFN‐α in the *Tatdn1* ^*−/−*^ heart (Fig. 5A). These GSEA Hallmark gene sets, related to biological processes, are characterized by the inclusion of an elevated proportion of genes coding for extracellular and integral membrane proteins (Data S1). We did not find Hallmark gene sets differentially expressed in the brain cortex at the selected statistical stringency. For each tissue, further analysis identified important enrichment in gene sets belonging to the C2 and C5 GSEA collections (curated and ontology, respectively), showing positive normalized enrichment scores (NES) (Fig. 5B). Annotated functions of these gene sets showed significantly high abundance of genes coding for proteins involved in transport across membranes, synapse formation, vesicle trafficking, cell adhesion, and cell–cell communication (Data S1). Plotting the top 50 genes upregulated and downregulated in each tissue ranked by fold change allowed to identify individual genes, which were most affected by *Tatdn1* deletion. Many of these gene code for extracellular proteins, membrane proteins such as receptors, channel subunits or signaling‐related proteins, and factors regulating the expression of genes involved in cell migration and axon growth (Fig. 5C). Microarray data validation was performed by individually analyzing four genes per tissue (two upregulated and two downregulated in the microarray) through RT‐qPCR. This analysis showed cardiac upregulation of *Slc22a17* and *Tmprss13*, which code for a cell surface receptor and a transmembrane protease, respectively, and downregulation of *Lama5*, which codes for a subunit of extracellular laminin. In the brain cortex, we confirmed upregulation of *Cd49a* and *Lag3*, which code for a cell surface glycoprotein and an inhibitory surface receptor, respectively, and downregulation of *Cbln4* and *F2rl2*, which code for a secreted protein and a surface receptor, respectively (Fig. S8). The identification of genes simultaneously regulated in the heart and brain showed 10 genes upregulated and 19 genes downregulated in both tissues (Fig. 5D; Data S1).

### Tatdn1 deficiency induces dilatation of the left cardiac ventricle

**Fig. 6:**
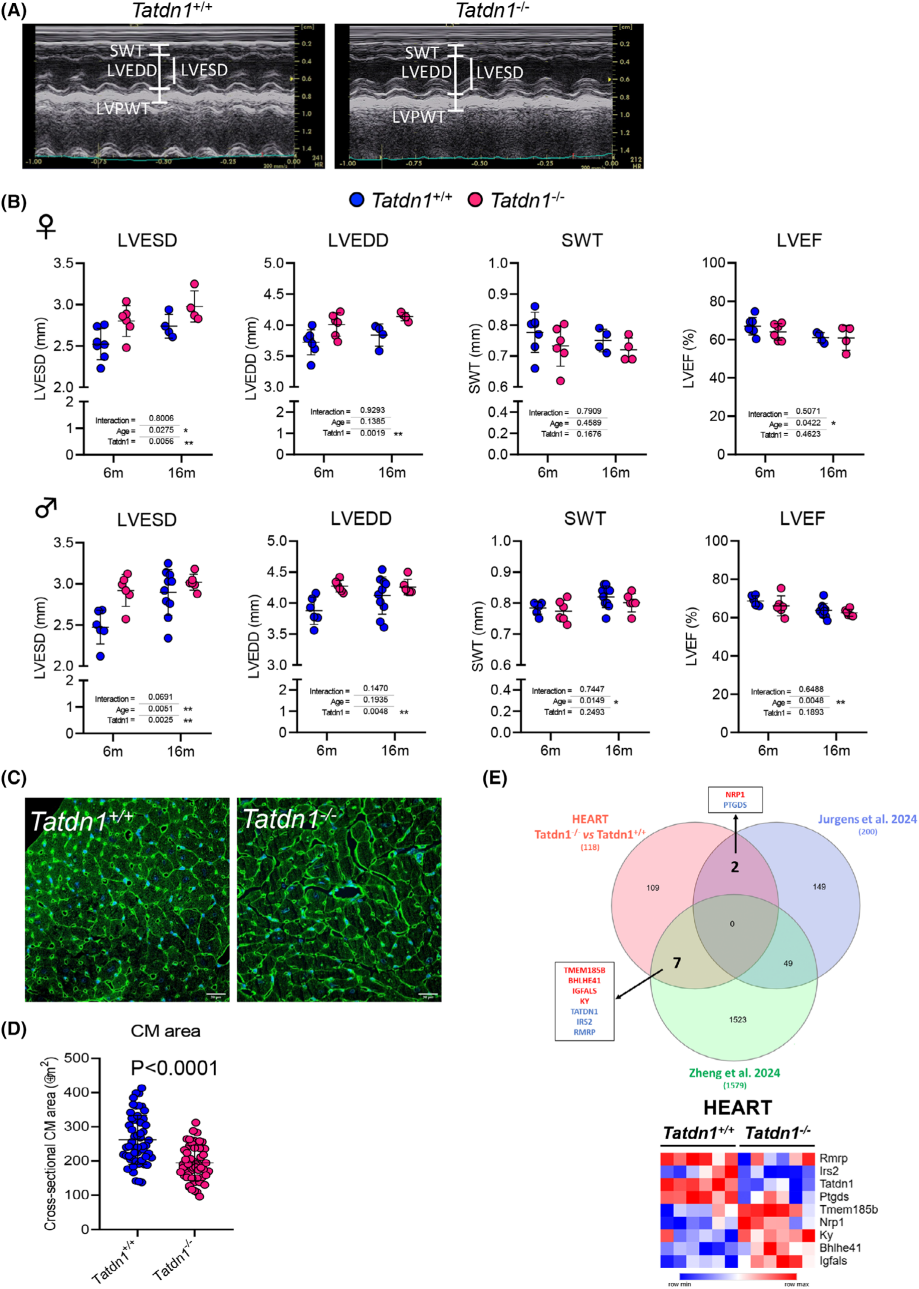
Adult TATDN1‐deficient mice display left ventricle dilatation and narrow cardiomyocytes. Six‐month‐old *Tatdn1* ^*+/+*^ (7 females, 6 males) and *Tatdn1* ^*−/−*^ (6 females, 6 males), and 16‐month‐old *Tatdn1* ^*+/+*^ (4 females, 10 males) and *Tatdn1* ^*−/−*^ (4 females, 6 males) mice were subjected to echocardiography. (A) Representative images of M‐mode echocardiographic images from six‐month‐old mice and (B) Main cardiac functional parameters directly measured or calculated from M‐mode data. LVEDD, left ventricular end‐diastolic diameter; LVESD, left ventricular end‐systolic diameter; LVEF, left ventricle ejection fraction; SWT, septum wall thickness. Individual values and mean ± SD are shown. (C) representative confocal microscopic images obtained from paraffin‐embedded myocardial sections from 6‐month‐old mice stained with FITC conjugated wheat germ agglutinin (green) and counterstained with Hoechst (blue, nuclei) (scale bar, 25 μm). (D) Short axis cardiomyocyte areas were measured (*n* = 54 cardiomyocytes/genotype, 3 mice per genotype, mean ± SD. Student's *t*‐test analysis). (E) Upper panel: Venn diagram showing differentially expressed genes (DEGs) shared across three datasets. The red circle represents DEGs in the heart of *Tatdn1* ^*−/−*^ *vs. Tatdn1* ^*+/+*^ mice (FC >2 or FC <0.5, *P* < 0.05, *n* = 118 genes at this stringency), the blue circle represents DEGs from Jurgens *et al*. [31] (*n* = 200), and the green circle represents DEGs from Zheng *et al*. [10] (*n* = 1579). Numbers within each section indicate the count of unique or shared DEGs. DEGs shared are highlighted, with genes in red showing upregulation and genes in blue showing downregulation. No DEGs were common to all three datasets. The diagram was generated using Interactivenn. Lower panel: Heatmap showing expression patterns of the selected DEGs in *Tatdn1* ^*−/−*^ *vs. Tatdn1* ^*+/+*^ mice hearts. The color scale represents normalized expression values, with red indicating higher expression and blue indicating lower expression.

*Tatdn1* is ubiquitously expressed in all organs (Fig. 1A). One of the organs with higher *Tatdn1* expression is the heart. Therefore, we decided to investigate the potential effects of *Tatdn1* deletion in the mouse heart. Echocardiographic characterization of adult *Tatdn1* ^*+/+*^ and *Tatdn1* ^*−/−*^ mice revealed cardiac dilatation at 6 months of age in *Tatdn1* ^*−/−*^ mice as reflected by increased left ventricular internal dimensions and a trend to reduced septum thickness (Fig. 6A,B). This cardiac alteration was associated with a slight reduction in contractile function although the difference related to the genotype was not statistically significant (Fig. 6B). Percent fractional shortening was normal in both genotypes at this age (32.6 ± 2.7 *vs*. 30.9 ± 3.1; *Tatdn1* ^*+/+*^ and *Tatdn1* ^*−/−*^, respectively) and the heart rate remained unaffected (468 ± 39 *vs*. 460 ± 51; *Tatdn* ^*1+/+*^ *vs. Tatdn1* ^*−/−*^ mean ± SEM), as summarized in Fig. S9. The same tendency was observed in 16‐month‐old animals. However, age‐related increased ventricular dilation, particularly during diastole, approached ventricular parameters and function of both genotypes (Fig. 6B; Fig. S9). Consistent with left cardiac ventricle dilation induced by *Tatdn1* deficiency, ventricular cardiomyocyte cross‐sectional area was smaller in six‐month‐old *Tatdn1* ^*−/−*^ mice compared to *Tatdn1* ^*+/+*^ (Fig. 6C,D).

A comparative analysis between our study on differentially expressed genes in the heart due to TATDN1 deficiency and two Gene‐wide association studies (GWAS) of human dilated cardiomyopathy [10, 31] identified nine genes, including TATDN1 [10], which were related to dilated cardiomyopathy in humans and in the TATDN1‐deficient mouse's ventricle (Fig. 6E). This gene set included those of transmembrane and secreted proteins (IRD2, TMEM185B, NRP1, and IGFALS) and genes involved in muscle growth and contraction (*PTGDS* and *KY*). Reducing stringency revealed 24 genes from which Ephrin Type A receptor 2 (EPHA2), involved in heart development and disease [32], was altered in the three studies (Fig. S10).

### Tatdn1‐deficient mice show specific alterations in locomotor activity and motor coordination

**Fig. 7:**
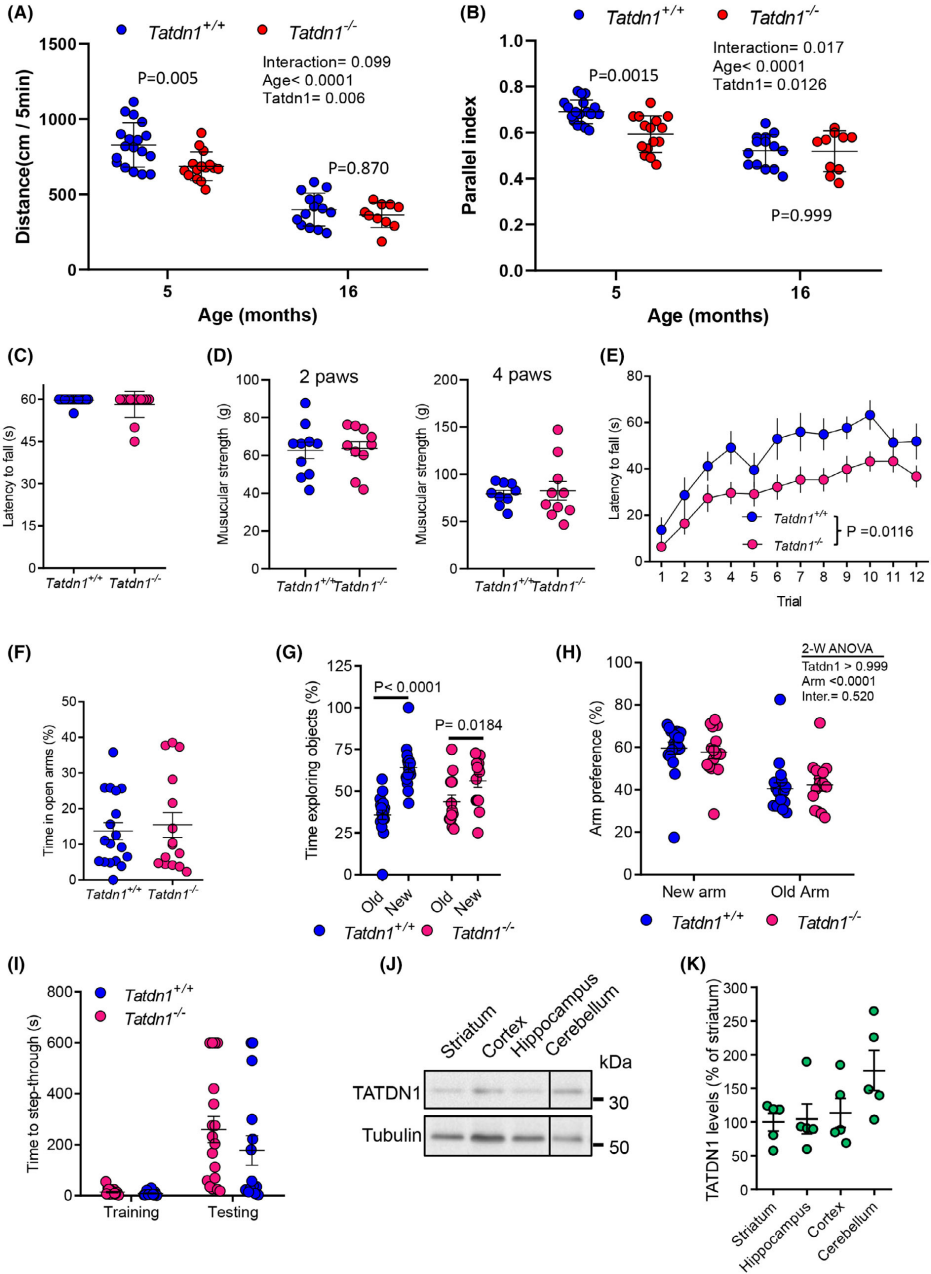
Behavioral characterization of TATDN1 deficient mice. Five‐month‐old adult *Tatdn1* ^*+/+*^ (8 females, 10 males) and *Tatdn1* ^*−/−*^ (8 females, 7 males) mice, and sixteen‐month‐old *Tatdn1* ^*+/+*^ (4 females, 10 males) and *Tatdn1* ^*−/−*^ (4 females, 6 males) mice were subjected to a comprehensive behavioral characterization. In the open field, (A) Locomotor activity and (B) Parallel index were monitored for 5 min. Locomotor activity, two‐way ANOVA, genotype effect *P* = 0.006. Parallel index, genotype effect *P* = 0.0126. (C) In the wire hanging test, the time until the mouse falls was measured in a 1‐min session. Mann–Whitney *t*‐test: sum of ranks A: 283, B: 213, Mann–Whitney U: 108, *P* = 0.347. (D) Muscular strength was also measured by using the automatized grip strength test. Muscular strength (measured in grams) was obtained from the forepaws (left) or from all four paws (right), *n* = 10/genotype. Student *t*‐test: *t* = 0.162, df = 17, *P* = 0.873 and *t* = 0.316, df = 11, *P* = 0.757, respectively. (E) Latency to fall was evaluated in the accelerating rotarod paradigm. Two‐way ANOVA, genotype effect: *F*_(1,31)_ = 7.194, *P* < 0.0116. (F) In the plus maze, the time spent in the open arms was monitored for 5 min in the two groups of mice. Student *t*‐test: *t* = 0.4234, df = 31; *P* = 0.6749. (G) In the novel object recognition test, recognition long‐term memory was evaluated 24 h after a training trial as the percentage of time exploring the new object *vs*. the old object. Two‐way ANOVA, genotype effect: *F*_(1,60)_ = 0.0001, *P* > 0.9999. (H) In the spontaneous alternation in a t‐maze, arm preference was evaluated 2 h after a training trial as the percentage of time exploring the new arm *vs*. the old arm. Two‐way ANOVA, genotype effect: *F*_(1,62)_ = 0.0001, *P* > 0.9999. (I) In the passive avoidance paradigm, the latency (seconds) to step‐through was evaluated in the training trial and in the testing trial 24 h after receiving an electric shock (2 s/1 mA). Two‐way ANOVA, genotype effect: *F*_(1,62)_ = 1.28, *P* = 0.2615. The same mice were used in all the behavioral tests (*n* = 18 *Tatdn1* ^*+/+*^ and 15 *Tatdn1* ^*−/−*^ five‐month‐old mice; *n* = 14 *Tatdn1* ^*+/+*^ and 10 *Tatdn1* ^*−/−*^ 16‐month‐old mice). For A–K, Mean ± SEM is depicted where appropriate. (J) Immunoblotting for TatD and tubulin as a loading control in the striatum, cortex, hippocampus, and cerebellum of 5‐month‐old *Tatdn1* ^*+/+*^ mice. (K) Densitometry quantification of TATDN1 protein levels.

Our next step on mice phenotype characterization was to evaluate the impact of TATDN1 deficiency in the brain and associated cognitive functions [33]. For this, adult *Tatdn1* ^*+/+*^ and *Tatdn1* ^*−/−*^ mice were subjected to a neurobehavioral test battery. First, we subjected the mice to the open‐field paradigm to evaluate spontaneous locomotion and exploration. Statistical analysis showed significant differences between groups in spontaneous locomotor activity being the five‐month‐old *Tatdn1* ^*−/−*^ mice hypoactive compared to control mice (Fig. 7A). In the same test, we also observed that *Tatdn1* ^*−/−*^ mice displayed a significant decrease on parallel index compared to *Tatdn1* ^*+/+*^ mice (Fig. 7B). This decrease indicated that *Tatdn1* ^*−/−*^ mice walked aberrantly less straight in contrast to the normal locomotion mode observed in healthy mice. Due to an age‐dependent reduction of spontaneous locomotion, the TATDN1‐dependent differences observed in these parameters were reduced in 16‐month‐old mice (Fig. 7A,B). These results indicated potential alterations in the motor systems in *Tatdn1* ^*−/−*^ mice. Therefore, we next aimed to evaluate muscular strength and motor coordination in the same mice. First, no significant differences were observed between genotypes in terms of muscular strength at the hanging wire (Fig. 7C). We then subjected five‐month‐old *Tatdn1* ^*+/+*^ and *Tatdn1* ^*−/−*^ mice to the automatized grip strength test and again we found no changes in muscular strength (Fig. 7D), reinforcing the idea that changes in locomotion and motor coordination were not due to putative muscular strength deficits. In contrast, *Tatdn1* ^*−/−*^ mice displayed reduced motor coordination in the accelerating rotarod task compared with *Tatdn1* ^*+/+*^ mice (Fig. 7E).

We then explored whether such alterations could be caused or associated with general neurologic impairments. To address this question, we first evaluated potential anxiety disturbances in *Tatdn1* ^*−/−*^ mice by using the plus maze test. In this test, no differences were observed between genotypes (Fig. 7F). Next, we evaluated different types of cognitive skills namely recognition memory, spatial alternation learning, and associative learning. Recognition memory was evaluated by using the novel object recognition test (Fig. 7G). In this test, both *Tatdn1* ^*+/+*^ and *Tatdn1* ^*−/−*^ mice showed equal increased preference for the new object indicating that this cognitive skill was spared in mutant mice. Regarding the spontaneous spatial alternation in the t‐maze, both *Tatdn1* ^*+/+*^ and *Tatdn1* ^*−/−*^ mice displayed similar rates of arm preference without significant differences between genotypes (Fig. 7H). Finally, associative learning measured in the passive avoidance paradigm was also similar between genotypes in terms of latency to step‐through after conditioning (Fig. 7I). In summary, behavioral measurements suggested a specific and consistent affectation of the locomotor and motor coordination skills in *Tatdn1* ^*−/−*^ mice compared to *Tatdn1* ^*+/+*^ mice.

We then aimed to study potential associations between TATDN1 expression patterns in the brain with the phenotype observed by the *Tatdn1* ^*−/−*^ mice. Interestingly, we observed that TATDN1 protein levels were enriched in the cerebellum (Fig. 7J,K). We next tried to analyze this specific expression pattern in tissue slices by immunofluorescence. However, despite the use of the same antibody to analyze the TATDN1 histological pattern in the Human Protein Atlas (see Materials section), our data showed identical unspecific staining in *Tatdn1* ^*+/+*^ and *Tatdn1* ^*−/−*^ cerebellum and heart tissues (Fig. S11). All data taken together indicate that TATDN1 in the brain could play a relevant role in cerebellar physiology that in turn is essential for an appropriate control of motor coordination and locomotion.

## Discussion

In this study, we present data exploring for the first time the distribution, function, and roles of TATDN1, the mammalian ortholog of bacterial TatD. Homologs of TatD are present in all kingdoms and have reported functions in DNA degradation [2, 16, 18] and protein quality control [15] in bacteria (*TatD*), roles in pathogen virulence [20, 21] in unicellular parasites (Plasmodium falciparum *PF3D7_0112000* and Trypanosoma brucei *Tb11.v5.0746*), and development in nematodes (*crn‐2*) [3]. Our results from four independent approaches demonstrate that TATDN1 is a cytoplasmic protein in mammalian cells. The ubiquitous expression of TATDN1 in mouse tissues, the lack of evidence of its role in DNA degradation, its induction during proteostasis stress along with the impairment of cardiac structure and motor control in *Tatdn1* ^*−/−*^ mice, suggest that TATDN1 plays a crucial role in cell biology, influencing organ function.

Several reports showed that bacterial TatD and some of its orthologs have the capacity to degrade DNA, so we first addressed the question whether TATDN1 is involved in DNA degradation using various strategies. However, at the levels expressed in cardiomyocytes and fibroblasts, TATDN1 does not appear to be involved in DNA degradation, or at least it is dispensable for this function, contrary to findings in bacteria, yeast, and protist *in vitro* recombinant systems [2, 18, 20, 21]. Supporting this, our results demonstrated that TATDN1 is cytosolic and does not translocate to the nucleus during cell death, contrary to what was described in the unicellular parasite *T. brucei* [21]. Regarding the previously reported nuclease activity of TatD, there is controversy over its substrate specificity and Mg^2+^ requirement. Some studies have shown that recombinant bacterial, yeast, and protist TatD homologs degrade double‐strand DNA (dsDNA) using linearized plasmids [2, 18], oligonucleotides [18] or nuclei [21] as substrates, while others reported that bacterial TatD preferentially cleaves single‐strand DNA (ssDNA) and RNA [16](summarized in Fig. S7). Notably, the latter would conflict with the role of TatD in DNA degradation during apoptosis. We evaluated the nuclease activity of endogenous TATDN1 in three models, including well‐characterized caspase‐independent [4, 5] and caspase‐dependent [34] cell death systems, as well as an *in vitro* assay [21] using cytosolic extracts at acid and basic pH to assess the potential effect of pH [18], but found no evidence of nuclease activity. Species‐specific differences in TatD homologs do not explain these discrepancies, as overexpressed TatD from *E. coli* has been shown to either cleave dsDNA [2] or be specific of ssDNA [16], depending on the study. Moreover, while bacterial and yeast TatD homologs apparently require Mg^2+^ to degrade DNA [2, 16, 18], the nuclease activity of overexpressed *P. falciparum* TatD‐like protein is inhibited by this cation [20]. An inhibitory effect of Mg^2+^ would also preclude a hypothetic DNase function within the cytosol or nucleus of mammalian cells, where free Mg^2+^ levels range from 1 to 5 mmol·L^−1^. Additionally, this would complicate the proposed role of *P. falciparum* TatD as an extracellular DNAse degrading NETs, given that human plasma Mg^2+^ levels are approximately 1 mmol·L^−1^. A potential explanation for TatD's role in NET degradation and parasite virulence, without directly degrading extracellular DNA, could be its involvement in the secretion of extracellular nucleases.

A recent study reported that recombinant human TATDN1 exhibited exonuclease activity *in vitro* on synthetic DNA [19]. The authors found that overexpressed human TATDN1 showed Mg^2+^‐dependent DNase activity, particularly on apurinic/apyrimidinic DNA, though with lower efficiency compared to other base excision repair enzymes like APE1 and EndoIV. Based on these *in vitro* assays, they hypothesized that TATDN1 functions as an endonuclease acting on apurinic DNA, potentially playing a role in DNA repair, and presumed its localization in the nucleus. However, they did not assess TATDN1 localization within cells or its DNase activity on chromatin DNA. Our findings, using three independent technical approaches, demonstrate that TATDN1 is cytosolic under both normal and stress conditions, and it does not participate in DNA degradation during apoptotic or nonapoptotic cell death. In our experiments, TATDN1 isolated from the cytosol showed no nuclease activity on linear dsDNA, suggesting that TATDN1 cytosolic role is unrelated to any DNase function or that its nuclease activity may be conditionally activated under specific circumstances.

Although TatD is dispensable for Sec‐independent protein export in bacteria [2], it has been implicated in the quality control of proteins secreted via the TatABC system [15]. In that study, the authors examined protein transport in *E. coli* strains lacking TatD (ΔTatD). Interestingly, the ΔTatD phenotype was not rescued by TatD overexpression, likely due to the imbalance in the TatABC transport system caused by abnormally high TatD levels [15]. Notably, while *P. falciparum* TatD was found to be required for extracellular NET degradation, this could be due to its role in extracellular nuclease secretion. However, the nuclease activity of *P. falciparum* TatD was analyzed under overexpression conditions [20]. In this context, our *in vivo* results suggest a potential role for mammalian TATDN1 in protein transport to membranes and the extracellular space. Total TATDN1 deficiency, achieved by crossing our *Tatd* ^*loxP/loxP*^ mice with a CAG‐*Cre* strain, led to changes in the expression of genes related to membrane functions and extracellular space (including ECM biology, receptor‐mediated signaling, channels, gap junction assembly) indicating that TATDN1 may be involved in these processes. In addition, we show that proteostatic stress induces TATDN1 expression, further suggesting its involvement in protein homeostasis.

The TatABC system is one of several alternative transport pathways found in bacteria and plant thylakoid membranes, responsible for secreting periplasmic proteins with an S‐R‐R motif in their N terminus [13]. Many of these proteins contain cofactors and must be secreted in a folded state. Although this transport system is absent in eukaryotic plasma membranes, it shares similarities with the eukaryotic Type I Unconventional Protein Secretion (UPS) system, which operates through transmembrane protein pores that are still poorly characterized [35]. The UPS system is particularly relevant during cellular stress and facilitates the secretion of factors involved in inflammation, immune response, and angiogenesis [36]. Interestingly, we observed induction of TATDN1 expression in two paradigms of proteostatic stress [26, 27] and gene expression changes in *Tatdn1* ^*−/−*^ hearts corresponded to GSEA gene sets for angiogenesis, inflammatory response, and IFN‐α response, including cytokines and growth factors. This suggests that the absence of TATDN1 may interfere with the normal function of the UPS system. Additionally, the epithelial mesenchymal transition GSEA gene set, which includes genes coding for proteins involved in ECM biology that rely on ER‐to‐Golgi transport, is also enriched in *Tatdn1* ^*−/−*^ hearts [37]. These significant changes in gene expression suggest a potential role for TATDN1 in the early stages of protein trafficking to membranes and the extracellular space, potentially impacting various transport systems.

The phenotype of *Tatdn1* ^*−/−*^ mice suggests that TATDN1 is not essential for basic biological functions, or that its roles may be compensated by other genes, at least under normal conditions. This is further supported by the lack of common enriched gene sets in the two tissues analyzed. Instead, TATDN1 appears to play tissue‐specific roles, consistently linked to functions involving membranes and the extracellular compartment. In TATDN1‐deficient mice, the heart shows early signs of dilated cardiomyopathy (DCM), characterized by an enlarged ventricle and thinner ventricular cardiomyocytes compared to controls. DCM is a leading cause of heart failure (HF), accounting for about 40% of HF cases in clinical trials [38]. Genetic factors are key contributors to DCM and influence the progression of non‐genetic DCM to HF [38]. Interestingly, a recent report associates *TATDN1*, among other genes, to human DCM [10]. Furthermore, Molecular Signatures Database (MSigDB) gene sets significantly altered in *Tatdn1* ^*−/−*^ hearts include angiogenesis, inflammatory response, and IFN‐α response, in agreement with gene expression changes previously observed in human DCM [39]. Additionally, TATDN1‐deficient hearts show altered expression of genes involved in cytoskeletal and sarcomeric protein interactions with the sarcolemma, ion flux, and cell–cell communication, which are also disrupted in human DCM [38, 40, 41]. Generation and analysis of conditional cardiac‐specific TATDN1‐deficient mice could provide further insights into the mechanisms underlying ventricular dilation and the potential development of ventricular dilation.

Analysis of gene expression and behavioral comparisons between *Tatdn1* ^*+/+*^ and *Tatdn1* ^*−/−*^ mice provided insights into the potential role of TATDN1 in the central nervous system. We observed that TATDN1 is most highly expressed in the cerebellum, a region critical for balance, motor coordination, and learning [42]. Accordingly, *Tatdn1* ^*−/−*^ mice displayed consistent and severe deficits in motor skills. Gene profiling of brain samples from *Tatdn1* ^*+/+*^ and *Tatdn1* ^*−/−*^ mice suggested that TATDN1 may regulate cerebellum‐dependent physiological and behavioral functions through genes enriched in this region, such as Calb2 [43] and Sema7a [44], both of which have been previously described to be implicated in motor coordination [45, 46]. Additionally, we cannot rule out the possibility that TATDN1 influences motor coordination through other brain regions involved in motor control such as the striatum [47], where TATDN1 is also expressed. Supporting this hypothesis, we found dysregulation of genes enriched in the striatum, including Adora2a [48], Adcy5 [49], and Egr3 [50], in *Tatdn1* ^*−/−*^ brains. These genes encode membrane‐bound and secreted proteins, suggesting that TATDN1 may be involved in the translation, folding, or export of these proteins. We propose that TATDN1 regulates molecular processes essential for motor coordination, particularly in brain regions such as the cerebellum.

In summary, we provide novel insights into the expression, distribution, and potential roles of TATDN1, the mammalian ortholog of *E. coli* TatD, the only conserved member of the bacterial tatABCD operon, with counterparts across all kingdoms. Our results demonstrate that mammalian TATDN1 is ubiquitously expressed and localized in the cytoplasm. Contrary to findings in overexpression systems and with recombinant proteins, our experiments do not support a role for TATDN1 in DNA degradation and suggest its implication in protein homeostasis. Given the importance of TATDN1 for proper heart development and motor coordination, as described here, further research is needed to elucidate its precise function and the molecular mechanisms underlying the phenotypes observed in TATDN1‐deficient mice.

## Limitations of the study

This study has several limitations that should be addressed in future research. (i) Although TATDN1 is conserved across all kingdoms and expressed in all cells and tissues analyzed, its potential housekeeping functions have not been experimentally explored. (ii) The observed posttranscriptional regulation of TATDN1 was not further investigated, which could provide additional insights into its functions. (iii) While nuclease activity has been tested against dsDNA in both cellular and *in vitro* contexts, we have not ruled out a potential role for TATDN1 in dsDNA nick formation or in the cleavage or degradation of ssDNA and/or RNA. (iv) We assessed TATDN1 nuclear translocation in two cell death paradigms, but further studies are needed across various cell types and conditions to confirm its consistent cytoplasmic localization. (v) Gene expression changes related to membrane functions, ECM biology, and ER‐to‐Golgi transport were among the most significant in *Tatdn1* ^*−/−*^ mice. In addition, we observed induction of TATDN1 expression during proteostatic stress and its implication in ER stress‐induced death. However, additional analysis is required to determine the specific molecular functions of TATDN1 in processes involved in protein biology. (vi) A comprehensive analysis of other organs and cell types is necessary to fully understand the impact of TATDN1 deficiency. (vii) Lastly, we did not investigate pathological conditions in *Tatdn1* ^*−/−*^ mice that might disrupt cell or tissue homeostasis. Identifying diseases where TATDN1 expression is altered could provide further insights into its relevance in mammals.

## Materials and methods

### Bioethics statement for Tatdn1 knockout mouse line and rat neonates

The investigation with experimental animals was approved by the Experimental Animal Ethic Committee (CEEA) of the University of Lleida (codes CEEA 05‐02/19, 06‐01/10, 07‐01/10, 08‐01/09 and 09‐01/09), complies with the ARRIVE Guidelines, and conforms to the Guide for the Care and Use of Laboratory Animals, 8th Edition, published in 2011 by the US National Institutes of Health [51]. The *Tatdn1* mice line with a C57BL/6J background was generated by the Australian Regenerative Medicine Institute (ARMI) at MONASH University has been crossed in our laboratory for several years with in house breed C57BL/6 J background mice and is housed in Tecniplast GM500 cages (391 × 199 × 160 mm) never exceeding 5 adults/cage. Rats used for neonatal cardiomyocyte culture had a Sprague–Dawley background and were breed and housed in conventional rooms. All animals were housed at the Experimental Animal Housing Facility—University of Lleida, lights on from 7 a.m. to 7 p.m., temperature = 18–22 °C, and 30–70% humidity. The enriched environment included autoclaved cellulose material. Animals were fed a 2914 diet (Irradiated Teklad Global 14% Protein Rodent Maintenance Diet, Harlan) and sterilized tap water, both *ad libitum*. Well‐being of animals is monitored daily by visual inspection and, for SPF‐housed mice, pathogen analysis is monitored from sentinel animals in periods of 8 weeks following the standards determined by the Federation of European Laboratory Animal Science Association (FELASA). Adult mice and neonatal rat pups were sacrificed following the Guidelines of our CEEA.

### Tatdn1 conditional knockout strategy and production of the Tatdn1^−/−^ mouse

The *Tatdn1* conditional knockout strategy and production of the floxed mouse was carried out by the Australian Regenerative Medicine Institute (ARMI) at MONASH University (Victoria, Australia), which also designed the procedure for Southern blot assessment and genotyping protocol. Briefly, *Tatdn1* exon 3 was flanked by *loxP* sites by electroporation of embryonic stem (ES) cells with a construct including the Neomycin cassette flanked by FRT sites. ES clones were selected and after Southern blot verification and cells were injected into blastocysts. Chimera males were crossed with C57BL/6 females to select germ line transmission, and *Tatdn1 loxP* allele‐carrying mice were bred to homozygosis. Homozygous *Tatdn1 loxP* ^*+/+*^ *Neo* ^*+/+*^ mice were crossed with *ROSA‐FLPe* mice (B6.129S4‐Gt(ROSA)26Sortm1(FLP1)Dym/RainJ, The Jackson Laboratory) to eliminate the Neomycin resistance cassette, and the homozygous descendants were crossed with *CAG‐Cre* mice (B6.Cg‐Tg(CAG‐Cre)CZ‐MO2Osb, RIKEN BRC) to get the whole body *Tatdn1* conditional knockout mouse. Since *Tatdn1* targeting did not affect mouse fertility, the *CAG‐Cre* transgene was eliminated from the breeding line by selecting *Cre* ^*neg*^ mice as progenitors. The procedure is described in detail in the Results section and Fig. S5. The *Tatdn1 loxP* ^*+/+*^ is deposited and available at the INFRAFRONTIER/EMMA repository (Neuherberg, Germany) with the code EM:13201 (*tatdn1* floxed).

### Cardiomyocyte counting and histological procedures

The hearts of neonatal mice were dissected, weighed, and cardiomyocytes were counted as previously described [28]. Briefly, hearts were dissected and formol‐fixed at 4 °C followed by a treatment with 12.5 mol·L^−1^ KOH overnight at 4 °C. After a 10 min vortex to dissociate the cells, they were passed through a 250 μm mesh, centrifuged, suspended in phosphate‐buffered saline (PBS) and cardiomyocytes were counted using a Neubauer chamber. Cardiomyocytes in the counting chamber were distinguished from other cellular types and debris by cytoplasmic size and cell shape. For the histologic analysis of adult hearts, mean cardiomyocyte cross‐sectional area was measured in 10 μm thick, short axis paraffin‐embedded myocardial sections from 3 adult mice per group. Sections were stained with FITC conjugated wheat germ agglutinin (WGA_FITC, L4895; Sigma, Burlington, MA, USA) to delineate the cell membrane, and nuclei were counterstained with 5 μg·mL^−1^ Hoechst 33342 (Invitrogen, Waltham, MA, USA). Images were obtained with a Zeiss LSM980 confocal microscope, and cardiomyocyte area was quantified using imagej software (Wayne Rasband, NIH, Bethesda, MD, USA).

### Cell lines

Human embryonic kidney cell line HEK293 (ATCC CRL‐1573; RRID:CVCL_0045) and Rat‐2 (ATCC CRL‐1764; RRID:CVCL_C8GZ) rat fibroblasts were purchased from the American Type Culture Collection and are continuously checked for Mycoplasma by PCR to keep the working stocks Mycoplasma‐free. All reagents for cell culture were from GIBCO (Thermo Fisher Scientific Inc., Waltham, MA, USA). All cell lines were grown in Dulbecco's modified Eagle medium (#41965–039) supplemented with 10% fetal bovine serum, 1 mmol·L^−1^ sodium pyruvate (#11360–039), 2 mmol·L^−1^ L‐glutamine (#25030‐024), 1 mmol·L^−1^ MEM NEAA (#11140‐035), and 100 U·mL^−1^ of penicillin/100 μg·mL^−1^ streptomycin (#15140‐122) at 37 °C with saturating humidity and 5% CO_2_. *Rps9* WT and D95N HEK293 clones were a kind gift from Prof. Böttger's Lab [26] (Institut fur Medizinische Mikrobiologie, Universitat Zurich, Switzerland). All experiments were conducted with low‐passage cells from recently resuscitated frozen stocks.

### Primary cell cultures

We obtained neonatal cardiomyocytes as previously described [52] from the heart of 2‐day‐old Sprague–Dawley rats or C57BL/6J mice using all available pups (usually 6–10 rat pups or 2–3 mouse pups per genotype), after digestion with type‐2 collagenase (Worthington, Lakewood, NJ, USA). We used a two‐round preplating to deplete the cardiomyocyte culture of nonmyocardial cells. Cells were plated at a density of 10^3^ cells/mm^2^ in 1 g·L^−1^ gelatin‐coated FALCON polystyrene dishes (Becton Dickinson, Palo Alto, CA, USA) and NUNCLON four‐well plates (NUNC, Denmark). The medium used was M199:DMEM 1:3 (Sigma‐Aldrich, Burlington, MA, USA), pH 7.2 with 7.2 mmol·L^−1^ glucose, 10% horse serum, and 5% fetal bovine serum (GIBCO, Carlsbad, CA, USA). Purity was assessed by fluorescent detection of α‐sarcomeric actinin (A7811; Sigma) visualized with an Olympus IX70 inverted epifluorescence and phase‐contrast microscope. Experimental ischemia was performed as reported elsewhere [4]. Briefly, cardiomyocyte cultures were washed with PBS and cultured in Tyrode's solution [53] (137 mmol·L^−1^ NaCl, 2.7 mmol·L^−1^ KCl, 8 mmol·L^−1^ Na_2_HPO_4_, 1.5 mmol·L^−1^ KH_2_PO_4_, 0.9 mmol·L^−1^ CaCl_2_, 0.5 mmol·L^−1^ MgCl_2_, initial pH 7.2) in the absence of serum, nutrients and buffering solution, during 12 h in a Whitley H35 Hypoxystation (Don Whitley Scientific) in a mixture of 5% CO_2_ and 95% N_2_ following manufacturer's instructions to attain a 0.2% oxygen concentration [8].

Neonatal dermal fibroblasts were obtained as described previously [34] from dorsal skin patches of the same pups from which cardiomyocytes were obtained. Dermal fibroblasts were cultured in DMEM medium plus 10% fetal bovine serum, 1 mmol·L^−1^ sodium pyruvate (#11360‐039), 1 mmol·L^−1^ MEM NEAA (#11140‐035), and 100 U·mL^−1^ of penicillin/100 μg·mL^−1^ streptomycin (#15140‐122) and used after 2–3 passages. Staurosporine (Sigma‐Aldrich, 19‐123) and the cell‐permeable pan‐caspase inhibitor Q‐VD‐OPh (quinolyl‐valyl‐O‐methylaspartyl‐[2,6‐difluorophenoxy]‐methyl ketone; Abcam 141 421, Cambridge, UK) were diluted in DMSO at a concentration of 1 mmol·L^−1^ in stock solution and kept frozen at −20 °C. A working dilution of 1 μmol·L^−1^ Staurosporine (STS) and 5 μmol·L^−1^ Q‐VD‐OPh (QVD) was achieved by adding the corresponding volume of stock to the culture medium (equal volume of DMSO was added to control plates) for the incubation times indicated in the figure legends.

### Cultured cardiomyocyte size measurement

Cardiomyocytes from *Tatdn1* ^*+/+*^ and *Tatdn1* ^*−/−*^ neonatal mice hearts were obtained as in Blasco *et al*. [7], seeded as indicated for rat cardiomyocytes, fixed, and stained for α‐actinin. Four to six microscopic images per genotype were recorded at a resolution of 4080 × 3072 pixels with an Olympus IX71 inverted fluorescence microscope and U‐RFL‐T power supply system (20× objective and 10× ocular magnifications plus 1.5× optical enhancement). Cross‐sectional areas of approximately 50 cells/condition were measured using the imagej software (Wayne Rasband, NIH). In this setting, 1 μm^2^ occupies 225 pixels. Cardiomyocyte cross‐sectional areas were transformed from pixels to μm^2^.

### Subcellular fractionation

Subcellular fractionation of primary neonatal cardiomyocytes from *Tatdn1* ^*+/+*^ and *Tatdn1* ^*−/−*^ hearts was performed as previously described [7] with the Subcellular Protein Fractionation kit for cultured cells (78 840; Thermo Fisher Scientific Inc.) following the manufacturer's guidelines. In addition, nuclear fraction was obtained with the Nuclei EZ Prep Nuclei Isolation kit (NUC101; Sigma‐Aldrich) which includes a gentle and nonionic detergent, Igepal CA‐630 (Sigma‐Aldrich), following supplier's instructions. Protein extraction, quantification, and analysis were performed as indicated below.

### Analysis of gene and protein expression

Total RNA extraction from cell cultures using the RNeasy Mini Kit (Qiagen, 74 104, Venlo, Netherlands) and reverse transcription was performed as described [28], and quantitative real‐time PCR was performed in an iCycler iQ PCR detection system and iQ v.3 and iQ v.5 software (Bio‐Rad Hercules, CA, USA), using the TaqMan Gene Expression Master Mix (4 369 016; Applied Biosystems, Waltham, MA, USA) and specific Gene Expression Assays from Applied Biosystems to amplify the transcript of mouse *Tatdn1* (Mm00613250_m1). Gene expression calculations were carried out as described previously for other genes [28]. Protein expression was analyzed in total protein extracts from tissues, cell cultures, and subcellular fractions diluted in Tris‐buffered 2% Sodium Dodecyl Sulfate (SDS) solution at pH 6.8, and SDS/PAGE and western blot were performed as described elsewhere [4, 5, 7]. Antibodies were against TATDN1 (Sigma, HPA023634, immunogenic sequence: CGEFEKNNPDLYLKELLNLAENNKGKVVAIGECGLDFDRLQFCPKDTQLKYFEKQFELSEQTKLP, used in the Human Protein Atlas, https://www.proteinatlas.org/ENSG00000147687‐TATDN1/summary/antibody), α‐actinin (Sigma, A7811), α‐tubulin (Sigma, T5168), Cytochrome c (Cell Signaling, 4272, Danvers, MA, USA), Cytochrome oxidase‐IV (Thermo Fisher, A21348), FLAG (Sigma, F3165), GAPDH (Abcam, ab8245), Lactate dehydrogenase (Rockland, 200‐1173‐0100, Philadelphia, PA, USA), Lamin A/C (BD Biosciences, 612 162, Franklin Lakes, NJ, USA). Secondary antibodies were mouse, rabbit, and goat IgG HRP (Sigma A9044, A0545, and A5420, respectively).

### TATDN1 overexpression

The coding sequence of *Mus musculus Tatdn1* contained in the mouse cDNA clone MGC:74327 IMAGE:30277245 (MRC Gene Service) was PCR‐amplified flanked by the BamHI and EcoRI sites and was subcloned into the pCDNA3.1 expression plasmid (Invitrogen) in frame and downstream to a 3×FLAG tag. The HEK293 cell line was transfected with the Lipofectamine reagent (Invitrogen) and overexpressed FLAG‐TATDN1 was detected by immunofluorescence and western blot with a monoclonal mouse anti‐FLAG antibody (Sigma, F3165). For immunofluorescence, cell nuclei were stained with Hoechst 33342 (Thermo Fisher, H1399).

### Proteomics‐based subcellular fractionation data analysis

Proteomics information about TATDN1 location, as well as the overall distribution of markers of cytosol, plasma membrane, mitochondria, and nucleus, was retrieved from the Martinez‐Val *et al*. publication [24]. There, HeLa and mouse samples (liver and muscle) were sequentially lysed to retrieve the protein content of six distinct subcellular compartments, which were further analyzed by liquid chromatography coupled to mass spectrometry. Characterization of each subcellular compartment was performed based on the annotation of known cell compartment markers, using the pRoloc R package [54]. The mass spectrometry proteomics data associated is available through the ProteomeXchange Consortium via the PRIDE partner repository [55] with the dataset identifier PXD023690.

### Lentiviral‐driven Tatdn1 gene silencing

*Tatdn1* gene silencing in rat cardiomyocytes and fibroblasts was achieved by transduction of lentiviral particles containing a *Tatdn1*‐specific small hairpin RNA construct or a scrambled sequence that was produced in HEK293T cells as detailed previously for other genes [4, 5, 6]. The silencing sequence for the *Tatdn1* rat transcript (XM_032888693) was: 5‐GATTGACTTGGACCTTTAT‐3′. Silencing efficiency was checked by western blot in each experiment.

### DNA integrity assay

DNA integrity in cell cultures was analyzed as previously described [4]. Briefly, cells were pelleted at the end of each treatment and frozen at −80 °C. Pellets from the same experiment were processed at once. They were diluted in 40 μL of sterile phosphate‐buffered saline, mixed with 40 μL of melted 1% low melting agarose (Sigma) in 0.5× Tris/Borate/EDTA (TBE) buffer (45 mmol·L^−1^ Tris pH 8.3, 45 mmol·L^−1^ boric acid, 1.0 mmol·L^−1^ EDTA). Each mixture was poured into a block caster and let to solidify. Each agarose block was submerged into 1 mL of lysis buffer (1% lauryl sarcosil, 0.5 mol·L^−1^ EDTA, 10 mmol·L^−1^ Tris, pH 8, 100 μg·mL^−1^ proteinase K) at 50 °C during 24 h in mild agitation and rinsed twice with 0.5× TBE for 1 h at room temperature. For analysis of DNA high molecular weight degradation, the blocks were then laid into wells of a 1% agarose, 0.5× TBE gel (Contour‐clamped homogeneous electric field (CHEF) grade, Sigma). Pulse field electrophoresis was performed in a CHEF DR‐II system (Bio‐Rad) set to the following protocol: run time, 14 h; switch time from 5 to 50 s; voltage gradient, 6 V/cm. Initial lysis buffer was further processed to analyze DNA low molecular weight degradation. Briefly, 1 mL of lysis buffer from DNA extraction was mixed with 1 mL of ethanol, kept at −20 °C for 18 h, and centrifuged. The pellet was rinsed with 70% ethanol, centrifuged again, and the final DNA pellet was diluted in 20 μL of 10 mmol·L^−1^ Tris, pH 8, 1 mmol·L^−1^ EDTA, and 10 μg·mL^−1^ RNase. Conventional 2% agarose gel electrophoresis was performed. Gels were stained with SYBR Safe (Molecular Probes, Eugene, OR, USA), visualized by UV exposure, and recorded with a Kodak DC290 digital camera.

### Nuclease activity *in vitro* test

Cytosolic extracts were obtained from *Tatdn1* ^*+/+*^ and *Tatdn1* ^*−/−*^ dermal fibroblasts with a buffer containing 220 mmol·L^−1^ mannitol, 70 mmol·L^−1^ sucrose, 10 mmol·L^−1^ KCl, 5 mmol·L^−1^ EGTA, and 2 mmol·L^−1^ MgCl_2_, pH 5, as described previously [52]. For the *in vitro* nuclease assay, we followed a protocol published for assessing recombinant TatD nuclease activity [21]. Briefly, freshly prepared extracts (10–30 μg of protein) were incubated with 1 μg of pcDNA3.1 plasmid (Invitrogen) linearized with BglII (TaKaRa, 1021A) and eluted in a buffer containing 5 mmol·L^−1^ Tris/HCl pH 8.5 in incubation buffer (25 mmol·L^−1^ MgCl_2_, 250 mmol·L^−1^ Tris/HCl final pH 8 or 6) to a final concentration of 5 mmol·L^−1^ MgCl_2_ and 50 mmol·L^−1^ Tris at pH 8 or 6, in a volume of 30 μL for 2 h at 37 °C. At the end of the incubation time, samples were run in 1% agarose gels stained with SYBR Safe (Molecular Probes), visualized by UV exposure, and recorded with a Kodak DC290 digital camera.

### Microarrays

Expression microarray experiments were designed and performed by the Unitat d'Estadística I Bioinformàtica (UEB) (Vall d'Hebron Institut de Recerca VHIR, Barcelona, Spain) with the Study Identification Number D1967. For microarrays, RNA was extracted using the RNeasy Mini Kit (Qiagen) from the cardiac left ventricle and cerebral cortex of either six *Tatdn1* ^*+/+*^ or *Tatdn1* ^*−/−*^ 2‐month‐old mice (three males and three females/genotype, totaling 24 samples). Samples passed several quality controls as scheduled by the facility. The microarray plate used was the Mouse Clariom S HT array (Applied Biosystems, Thermo Fisher, 902 971). A detailed description of the procedures and raw microarray data are available at the NCBI's Gene Expression Omnibus (GEO) database (GEO Series record GSE167387).

### Bioinformatics analysis of microarray data

Microarray data from the twenty‐four CEL Affymetrix files was used to identify Gene signatures determined with GSEA version 4.0.1 (Broad Institute, Cambridge, MA, USA) using the hallmark gene sets, the C2 curated gene sets, the C5 gene ontology gene sets, and the C6 oncogenic signatures (Molecular Signature Database v2.5). A two‐class analysis with 1000 permutations of gene sets and a weighted metric was used. Expression heatmaps of angiogenesis, epithelial mesenchymal transition, and interferon alpha response genes were created using Morpheus software (https://software.broadinstitute.org/morpheus/, Broad Institute). A complete list of enriched gene sets is provided in Data S1. Venn diagrams were performed using the online software tool InteractiVenn [56] (https://www.interactivenn.net/index2.html).

To examine whether the identified best‐scored genes from GSEA are meaningful to biological processes, we conducted gene set enrichment analysis through the ‘Investigate Gene Sets’ function of the web based GSEA software tool (http://www.broadinstitute.org/gsea/msigdb/annotate.js) [57].

### Transthoracic echocardiography

Transthoracic echocardiography was performed using a Vivid Q portable ultrasound system equipped with an i12L‐RS 13 MHz transducer (GE Healthcare) as described earlier [58]. The end‐diastolic left ventricular internal diameter (LVEDD) and volume (LVEDV), end‐systolic left ventricular internal diameter (LVESD) and volume (LVESD), interventricular septum thickness (IVS) and posterior wall thickness (LVPW) at end diastole were measured in M‐mode recordings. Ejection fraction (EF) and fractional shortening (FS) were calculated according to standard formulas. An investigator blinded to the groups analyzed the images off‐line. Thirteen six‐month‐old *Tatdn1* ^*+/+*^ (6 males, 7 females) and 12 *Tatdn1* ^*−/−*^ mice (6 males, 6 females) and 14 sixteen‐month‐old *Tatdn1* ^*+/+*^ (4 females, 10 males) and 10 *Tatdn1* ^*−/−*^ (4 females, 6 males) mice were used. For each parameter, measurements were performed from three to six different cardiac cycles, and the values were averaged.

### Behavioral assessment

Five‐month‐old *Tatdn1* ^*+/+*^ (8 females, 10 males) and *Tatdn1* ^*−/−*^ (8 females, 7 males), and 16‐month‐old *Tatdn1* ^*+/+*^ (4 females, 10 males) and *Tatdn1* ^*−/−*^ (4 females, 6 males) mice were subjected to a comprehensive behavioral characterization as previously described [33, 59, 60]. To check spontaneous locomotor activity, we used the open field. Briefly, the apparatus consisted of a white square arena measuring 40 × 40 × 40 cm in length, width, and height, respectively. The dim light intensity was 60 lux throughout the arena. Animals were placed in the arena center and allowed to explore freely for 5 min. Spontaneous locomotor activity and parallel index (deambulation patterns, circular *vs*. straight) were measured. At the end of each trial, any defecation was removed, and the apparatus was wiped with 30% ethanol, as previously described [61]. Animals were tracked and recorded with SMART junior software (Panlab).

To analyze mouse anxiety, we used the elevated plus‐maze paradigm. Briefly, the plus‐maze was made of plastic and consisted of two opposing 30 × 8 cm open arms and two opposing 30 × 8 cm arms enclosed by 15‐cm‐high walls. The maze was raised 50 cm from the floor and lit by dim light. Each mouse was placed in the central square of the raised plus‐maze, facing an open arm, and its behavior was scored for 5 min. We recorded the time spent in the open arms, which normally correlates with low levels of anxiety. Animals were tracked and recorded with SMART junior software (Panlab).

Muscular strength and neuromuscular abnormalities were analyzed by the wire hanging test. A standard wire cage lid was used. To test balance and grip strength, mice were placed on top of a wire cage lid. The lid was shaken slightly to cause the mouse to grip the wires, and then, it was turned upside down for 60 s. The number of falls and latency to fall of each mouse was recorded. To complete the muscular strength assessment, a computerized grip strength meter (Almemo 2450, Ahlborn) was used. We performed three measures for forepaws in one hand and three measures for all four paws together on the other hand. Each measure was considered a replicate, and a mean from these three measures was obtained.

For motor coordination and learning, we used the accelerating rotarod task. In this test, animals were placed in the motorized rod (30‐mm diameter, Panlab, Spain). The rotation speed gradually increased from 4 to 40 rpm. over the course of 5 min. The fall latency time was recorded when the animal was unable to keep up with the increasing speed and fell. Rotarod training/testing was performed 4 times per day for 3 consecutive days. The results show the average fall rates per trial for group each day.

The T‐maze apparatus used for the T‐SAT (T‐spontaneous alteration task) was a wooden maze consisting of three arms, two of them situated at 180° from each other, and the third, representing the stem arm of the T, situated at 90° with respect to the other two [61]. All arms were 45 cm long, 8 cm wide and enclosed by a 20 cm wall. The maze was thoroughly painted with waterproof gray paint. Light intensity was 5 lux throughout the maze. A 10 cm start area was situated at the end of the stem arm and closed by a wooden guillotine door. Two identical guillotine doors were placed in the entry of the arms situated at 180°. In the training trial, one arm was closed (novel arm) and mice were placed in the stem arm of the T (home arm) and allowed to explore this arm and the other available arm (familiar arm) for 10 min, after which they were returned to the home cage. After inter‐trial intervals of 1 h, mice were placed in the stem arm of the T‐maze and allowed to freely explore all three arms for 5 min, as previously reported [61]. The arm preference was determined by calculating the time spent in each arm × 100/time spent in both arms (familiar/old and novel/new arm).

In the novel object recognition test (NORT), the device consisted of a white square arena measuring 40 × 40 × 40 cm in length, width, and height, respectively. The light intensity was 60 lux throughout the arena, and the room temperature was kept at 19–28 °C and 40–60% humidity. Mice were initially habituated to the arena in the absence of objects (1 day, 30 min). On the second day, two similar objects were presented to each mouse during 10 min (A′A″ condition) after which they were returned to the home cage. Twenty‐four hours later, the same animals were retested for 5 min in the arena with a familiar and a new object (A′B condition). The object preference was measured as the time exploring each object × 100/time exploring both objects. The arena was rigorously cleaned between animal trials to avoid odors. Animals were tracked and recorded with SMART junior software (Panlab).

For the passive avoidance (light–dark) paradigm, we conducted the experiments in a 2‐compartment box, where 1 compartment was dimly lit (20 lux) and preferable to a rodent and the other compartment was brightly lit (200 lux); both chambers were connected by a door (5 cm × 5 cm). During training, mice were placed into the aversive brightly lit compartment; and upon entry into the preferred dimly lit compartment (with all 4 paws inside the dark chamber), mice were exposed to a mild foot shock (2 s foot shock, 1 mA intensity). The latency of mice to enter the dark chamber was recorded. Twenty seconds after receiving the shock on the foot, mice were returned to the home cage until testing. After 24 h (long‐term memory), animals were tested for retention. In the retention test, mice were returned to the brightly lit compartment again, and the latency to enter the shock‐paired compartment (dark chamber) was measured (retention or recall latency). Ten minutes was used as a time cutoff in the retention test.

### Statistics

Statistical analysis of data was performed using the GraphPad Prism software (GraphPad Prism Software version 8.0.1, Boston, MA, USA). The nonparametric Mann–Whitney U‐test was used when comparing one variable in two independent data groups (Fig. 3E–G), and two‐way ANOVA was used when comparing two variables followed by Sidak's *post hoc* test for multiple comparisons (Figs 1 and 3). Echocardiographic parameters were compared using ANOVA and Student *t*‐test, as indicated in the figure legend (Fig. 6B,D). In the behavior experiments, parametric Student *t*‐test and two‐way ANOVA were applied, as indicated in the figure legend, as previously reported. The exact number of independent measurements, replicates, and statistical values are specified in each figure. All statistical tests were two‐sided at a significance level of <0.05.

## Conflict of interest

All authors declare no conflicts of interest.

## Author contributions

Conceptualization, DS; methodology, JG‐V, JI, PP‐G, MR‐M, AG, JVO, ML, and DS; investigation, GB, AB, AS‐B, JGV, DA, SG‐C, EM‐C, SB‐P, SH, AM‐V, and FT; validation, all authors; formal analysis, JG‐V, JI, AG, ML, and DS; resources, JGV, XC, JXC, PP‐G, and DS; writing—original draft preparation, DS with AG contribution for the neurobehavior section and AM‐V for the proteomics data analysis; writing—review and editing, DS with the contributions of all authors; supervision, DS; project administration, DS; funding acquisition, JI, JXC, MR‐M, PP‐G, AG, and DS.

## Peer review

The peer review history for this article is available at https://www.webofscience.com/api/gateway/wos/peer‐review/10.1111/febs.70077.

## Supporting information


**Fig. S1.** Information available about TatD homologues in several genome databases of model organisms.
**Fig. S2.** Alignment of TATD/TATDN1 sequence conservation across species.
**Fig. S3.** TATDN1 expression in cells of the heart and brain, and subcellular fractionation controls for the HeLa spatial proteomics analysis.
**Fig. S4.** Search result for TATDN1 protein using BarCode single protein localization app (https://lehtio‐lab.se/subcellbarcode/).
**Fig. S5.** Process for the conditional Tatdn1 allele deletion in mice.
**Fig. S6.** Body weight of adult Tatdn1^+/+^ and Tatdn1^−/−^ mice, and correction by tibial length.
**Fig. S7.** Summary of the most relevant published knowledge about TatD/TATDN1.
**Fig. S8.** Validation of microarray data from the heart and brain of Tatdn1^+/+^ and Tatdn1^−/−^ mice.
**Fig. S9.** Echocardiographic data complementing Fig. 6.
**Fig. S10.** Venn diagram illustrating differentially expressed genes (DEGs) shared across datasets of cardiac genes differentially expressed in Tatdn1^−/−^ *vs*. Tatdn1^+/+^ mice (this article), Jurgens *et al*., 2024 and Zheng *et al*., 2024.
**Fig. S11.** Immunofluorescence analysis of paraformaldehyde fixed cerebellar and cardiac tissues of Tatdn1^+/+^ and Tatdn1^−/−^ adult mice.

**Data S1.** Gene Set Enrichment Analysis (GSEA) data.

## Data Availability

All data supporting the findings of this study are available within this article and its supporting information. Additional requests will be provided upon demand. The *Tatdn1 loxP* ^*+/+*^ is deposited and available at the INFRAFRONTIER/EMMA repository (Neuherberg, Germany) with the code EM:13201 (*tatdn1* floxed). Detailed description of the procedures and raw microarray data are available at the NCBI's Gene Expression Omnibus (GEO) database (GEO Series record GSE167387).
