# Supplementary material for: Mammalian TatD DNase domain containing 1 (TATDN1) is a proteostasis‐responsive gene with roles in ventricular structure and neuromuscular function
Source: FEBS J. 2025 Mar 23;292(19):5105–29. doi: 10.1111/febs.70077 (PMC12505432; doi:10.1111/febs.70077)
Supplement: Supplementary file 1 — Fig. S1. Information available about TatD homologues in several genome databases of model organisms. Fig. S2. Alignment of TATD/TATDN1 sequence conservation across species. Fig. S3. TATDN1 expression in cells of the heart and brain, and subcellular fractionation controls for the HeLa spatial proteomics analysis. Fig. S4. Search result for TATDN1 protein using BarCode single protein localization app (https://lehtio‐lab.se/subcellbarcode/). Fig. S5. Process for the conditional Tatdn1 allele deletion in mice. Fig. S6. Body weight of adult Tatdn1+/+ and Tatdn1−/− mice, and correction by tibial length. Fig. S7. Summary of the most relevant published knowledge about TatD/TATDN1. Fig. S8. Validation of microarray data from the heart and brain of Tatdn1+/+ and Tatdn1−/− mice. Fig. S9. Echocardiographic data complementing Fig. 6. Fig. S10. Venn diagram illustrating differentially expressed genes (DEGs) shared across datasets of cardiac genes differentially expressed in Tatdn1−/− vs. Tatdn1+/+ mice (this article), Jurgens et al., 2024 and Zheng et al., 2024. Fig. S11. Immunofluorescence analysis of paraformaldehyde fixed cerebellar and cardiac tissues of Tatdn1+/+ and Tatdn1−/− adult mice. [file FEBS-292-5105-s002.pdf]

Supplementary Figure 1. Model organisms databases consulted for the presence of TatD-like genes:

E. Coli Database https://ecocyc.org/ECOLI/compare-frame-in-orgs?type=ORTHOLOG&object=EG11481&initial-orgs=ECOLI identifies Ec TatD as ortholog of Tatdn1 (Rn), Sc YBL055C and Ce crn-2.

Genecards (Human) https://www.genecards.org/cgi-bin/carddisp.pl?gene=TATDN1&keywords=tatdn1 identifies TATDN1 as ortholog of Dm CG3358, Ce crn-2, Sc YBL055C

The Eukaryotic Pathogen, Vector and Host Informatics Resource (VEuPathDB):
Plasmodium https://plasmodb.org/plasmo/app/record/gene/PF3D7_0112000
Trypanosoma https://tritrypdb.org/tritrypdb/app/record/gene/Tb11.v5.0746

WormBase https://wormbase.org/species/c_elegans/gene/WBGene00000795#0-9f36h1g-10 identifies crn-2 as ortholog of Tatdn1 and YBL055C

FlyBase http://flybase.org/reports/FBgn0033117 Identifies CG3358 as ortholog of Tatdn1, crn-2 and YBL055C

Saccharomyces Genome Database https://www.yeastgenome.org/locus/S000000151/protein identifies Sc YBL055C as ortholog of Tatdn1 (Hs, Rn, Mm), Ce crn-2, Dm CG3358

OrthoDB v10.1, a hierarchical catalog of orthologs
Entry for Tatdn1 https://www.orthodb.org/?level=&species=&query=2331935at2759
Lists 3438 genes as orthologs of the “TatD family” in 1894 species in eukaryotes, including C elegans crn-2, S cerevisiae YBL055C, D melanogaster CG3358; Protist Tatd-like (Plasmodium, Leishmania and Trypanosome species) and mammalian Tatdn1.

(a)

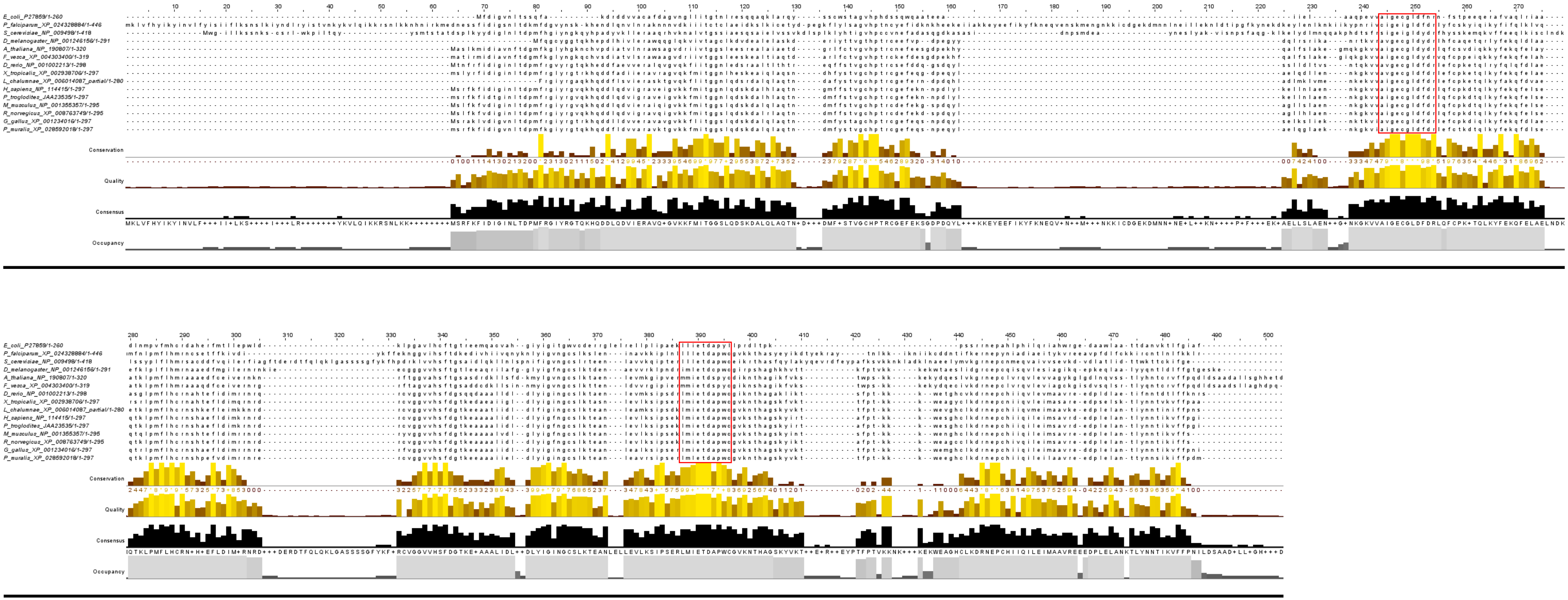

Supplementary Figure 2. Analysis of TATD/TATDN1 conservation across species. (a) Clustal alignment of TATD/TATDN1 sequences from Human, H sapiens_NP_114415; Chimpanzee, P troglodites JAA23535; Mouse, M musculus NP_001355357; Rat, R norvegicus XP_008763749; Zebrafish, D rerio NP_001002213; Coelacanth, L chalumnae XP_006014087_partial; Chicken, G gallus XP_001234016; Wall lizard, P muralis XP_028592018; Clawed frog, X tropicalis XP_002938706; Fruit fly, D_melanogaster NP_001246156; Thale cress, A_thaliana NP_190807; Strawberry, F vesca XP_004303400; Baker's yeast, S cerevisiae NP_009498; Malaria's plasmodium, P falciparum XP_024328884; E coli P27859; from animal, plant, protist, fungi and bacteria taxons. Red squares: highly conserved motifs.

Conservation is visualized on the alignment or a sequence group as a histogram giving the score for each column. Conserved columns are indicated by '*' (score of 11 with default amino acid property grouping), and columns with mutations where all properties are conserved are marked with a '+' (score of 10, indicating all properties are conserved). The quality score is calculated for each column in an alignment by summing, for all mutations, the ratio of the two BLOSUM 62 scores for a mutation pair and each residue's conserved BLOSUM62 score (which is higher). This value is normalized for each column, and then plotted on a scale from 0 to 1. The consensus displayed below the alignment is the percentage of the modal residue per column. If the modal value is shared by more than 1 residue, a "+" symbol is used in the display.

(b)

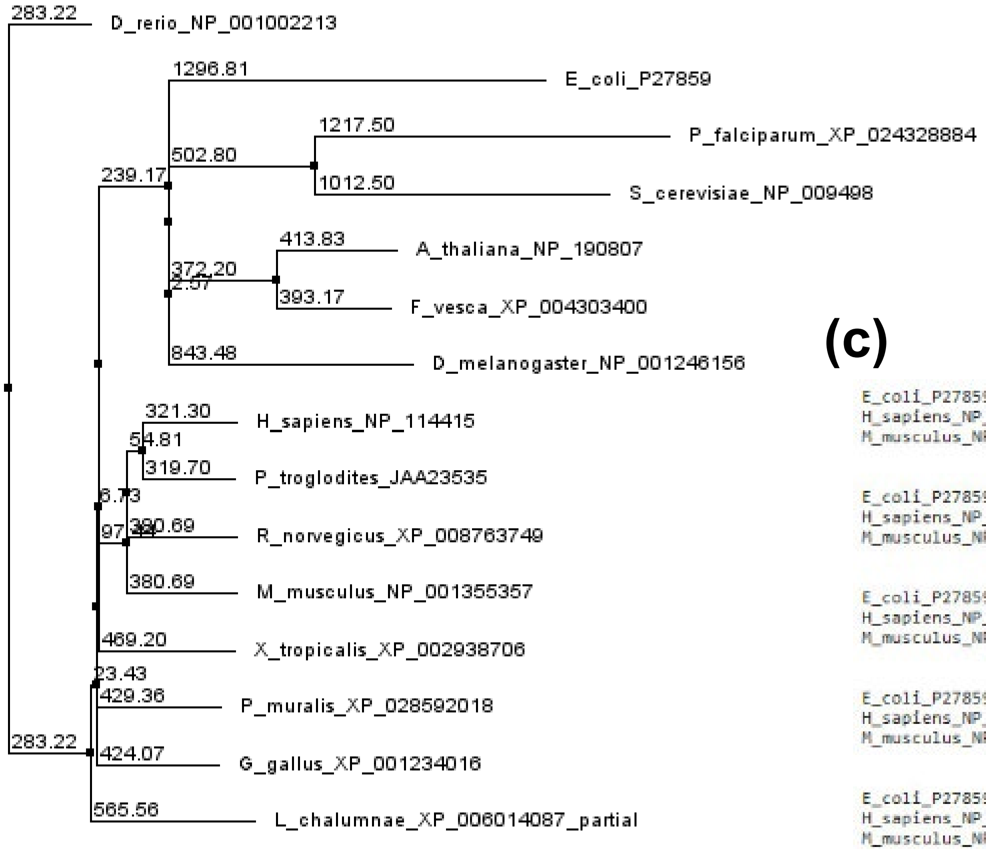

(c)

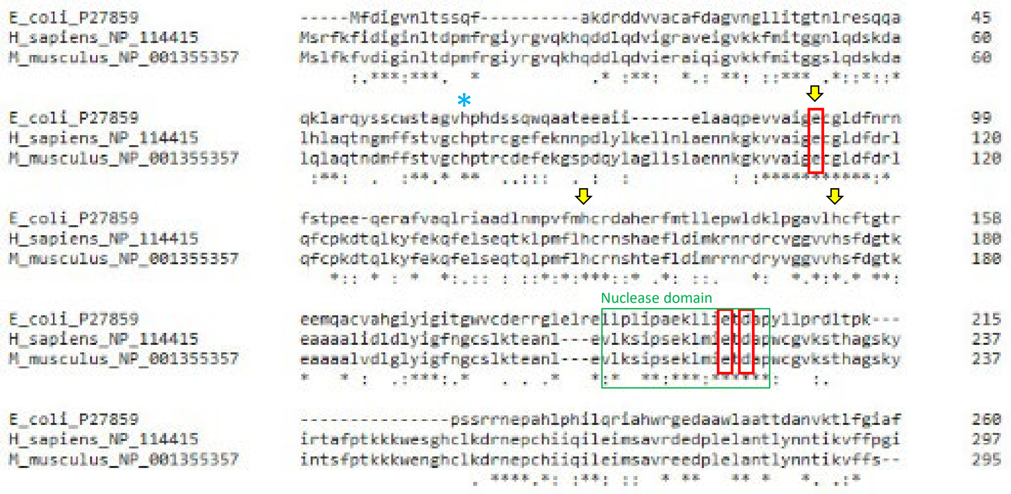

Supplementary Figure 2. Analysis of TATD/TATDN1 conservation across species. (b) Phylogenetic tree was automatically calculated from the aligned sequences. (c) Clustal alignment of *E coli* TatD protein and its human and mouse orthologs. Red squares highlight metal binding residues and blue asterisk shows the catalytic residue, as described in Chen et al. 2014 (https://doi.org/10.1093/nar/gku732) (see main text). Yellow arrows show metal binding residues and green square indicates the nuclease domain as shown in Uniprot (https://www.uniprot.org/uniprotkb/P27859/entry).

Alignment and tree were performed with Clustal Ω (https://www.ebi.ac.uk/Tools/msa/clustalo/) and visualized with Jalview 2.11.1.3 Waterhouse, A.M., Procter, J.B., Martin, D.M.A, Clamp, M. and Barton, G. J. (2009) Jalview Version 2 - a multiple sequence alignment editor and analysis workbench Bioinformatics doi: 10.1093/bioinformatics/btp033.

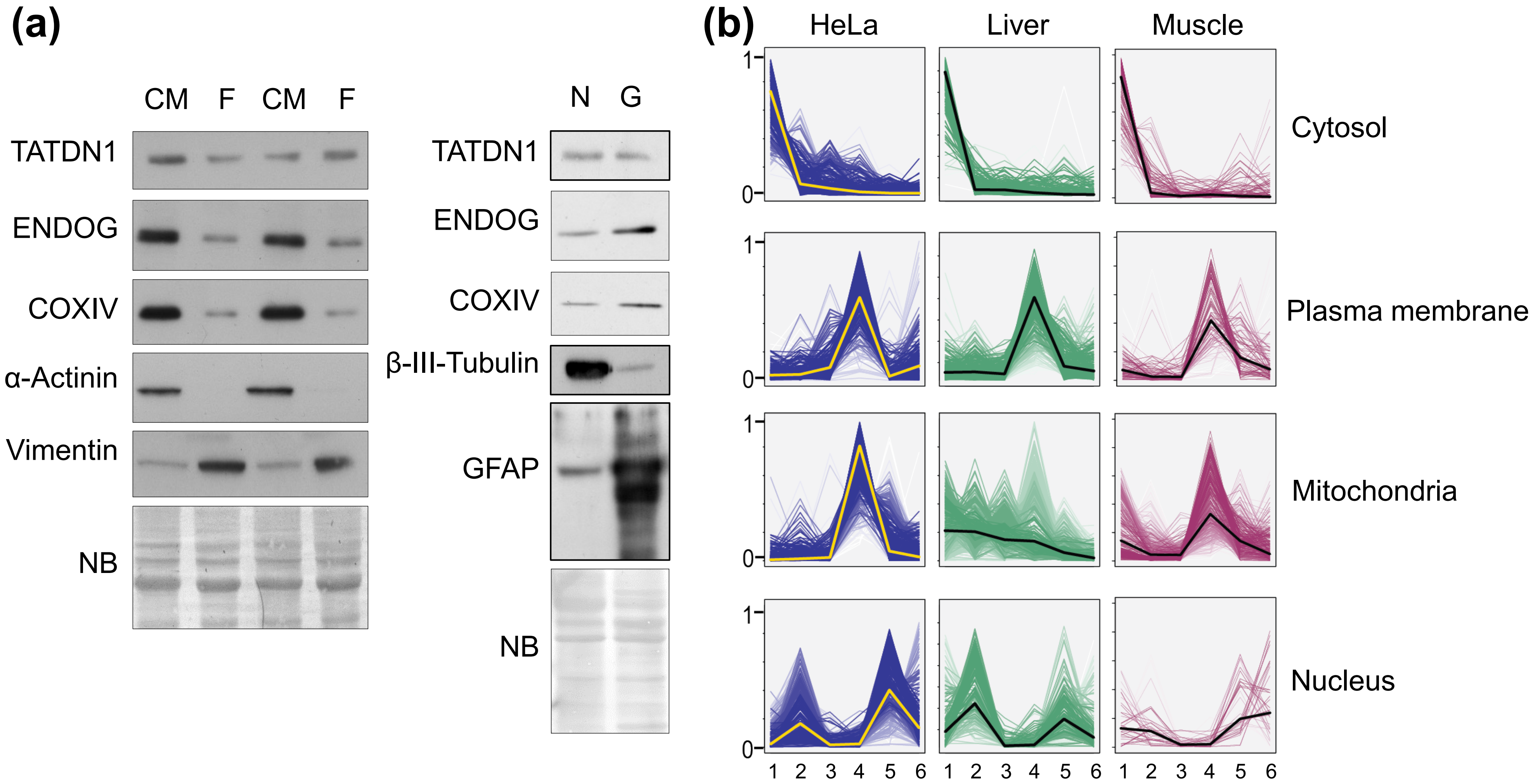

Supplementary Figure 3. TATDN1 expression in cells of the heart and brain, and subcellular fractionation controls for the HeLa spatial proteomics analysis. (a) Protein detection in total protein extracts of cultured neonatal rat ventricular cardiomyocytes (CM), cardiac fibroblasts (F), cortical neurons (N) and glia (G). Data shown are from two independent cardiomyocyte and fibroblast isolations and an isolation of cortical neurons and glia. TATDN1, ENDOG (Endonuclease G; mitochondria); COXIV (Cytochrome Oxidase Complex IV; mitochondria); α-Actinin (cardiac muscle); Vimentin (fibroblast); β-III-Tubulin (neuron); GFAP (Glial fibrilar acidic protein, glia); NB (Naphthol blue, protein staining). (b) Profile-plots of cell compartment markers retrieved from Gatto et al 2014 pRoloc package (PMID: 24413670) in the subcellular fractions obtained from HeLa, mouse liver and mouse muscle referent to figure 1e. Scaled intensity (from 0 to 1) across fractions 1-6 is plotted for each independent replicate. Gradient of color indicates Pearson correlation to the centroid of each distribution, which is highlighted as a yellow/black line.

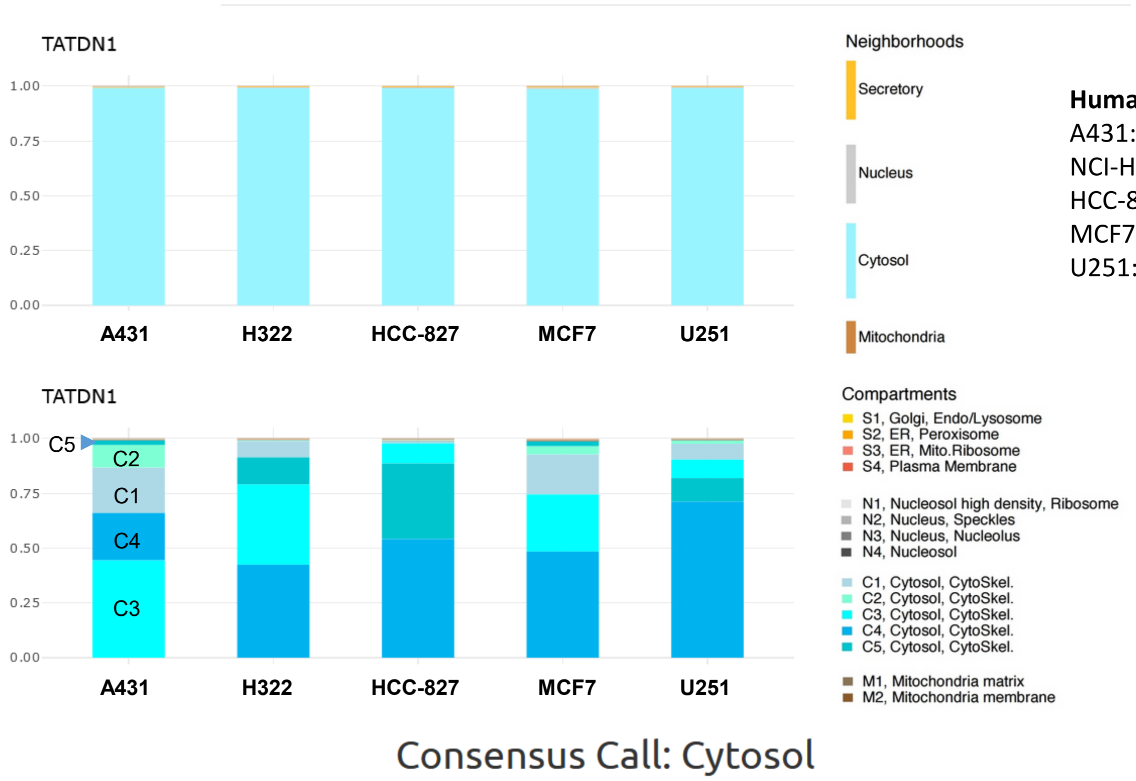

Human cancer cell lines
A431: epidermoid carcinoma
NCI-H322: lung cancer
HCC-827: lung cancer
MCF7: breast cancer
U251: glioblastoma

Supplementary Figure 4. Search result for TATDN1 protein using BarCode single protein localization app (https://lehtio-lab.se/subcellbarcode/). A mass spectrometry-based analysis of cellular subfractions from five human cancer cell lines (A431, NCI-H322, HCC-827, MCF7 and U251) detected endogenous TATDN1 protein in C1 to C5 cytosolic fractions. TATDN1 peptides were absent in nuclear fractions.

Orre, L. M., Vesterlund, M., Pan, Y., Arslan, T., Zhu, Y., Fernandez Woodbridge, A., Frings, O., Fredlund, E., & Lehtiö, J. (2019). SubCellBarCode: Proteome-wide Mapping of Protein Localization and Relocalization. Molecular Cell, 73(1), 166-182.e7. https://doi.org/10.1016/J.MOLCEL.2018.11.035

Supplementary File 1_Figure 5

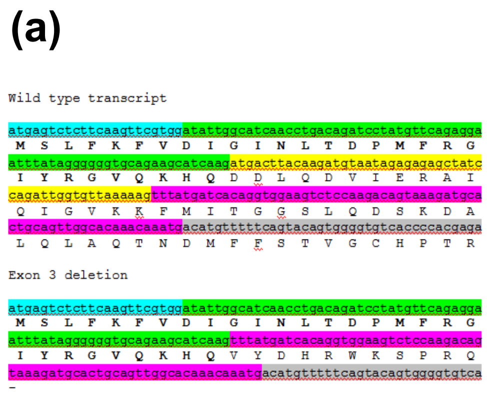

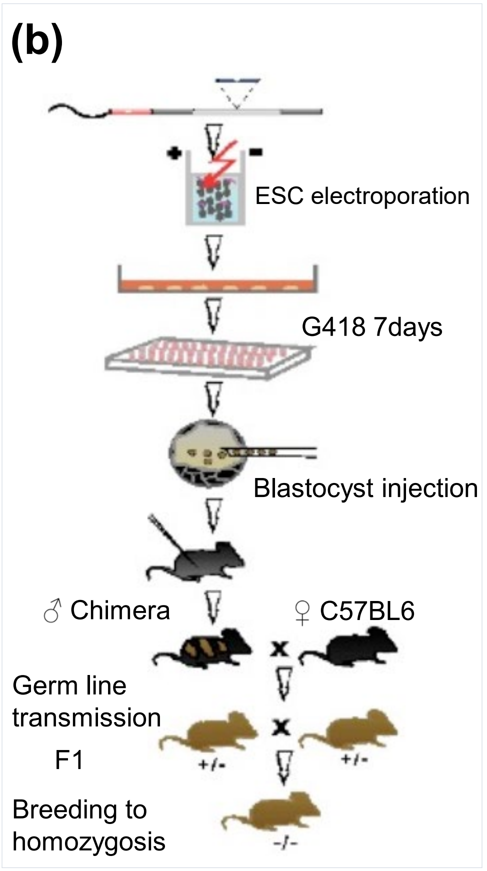

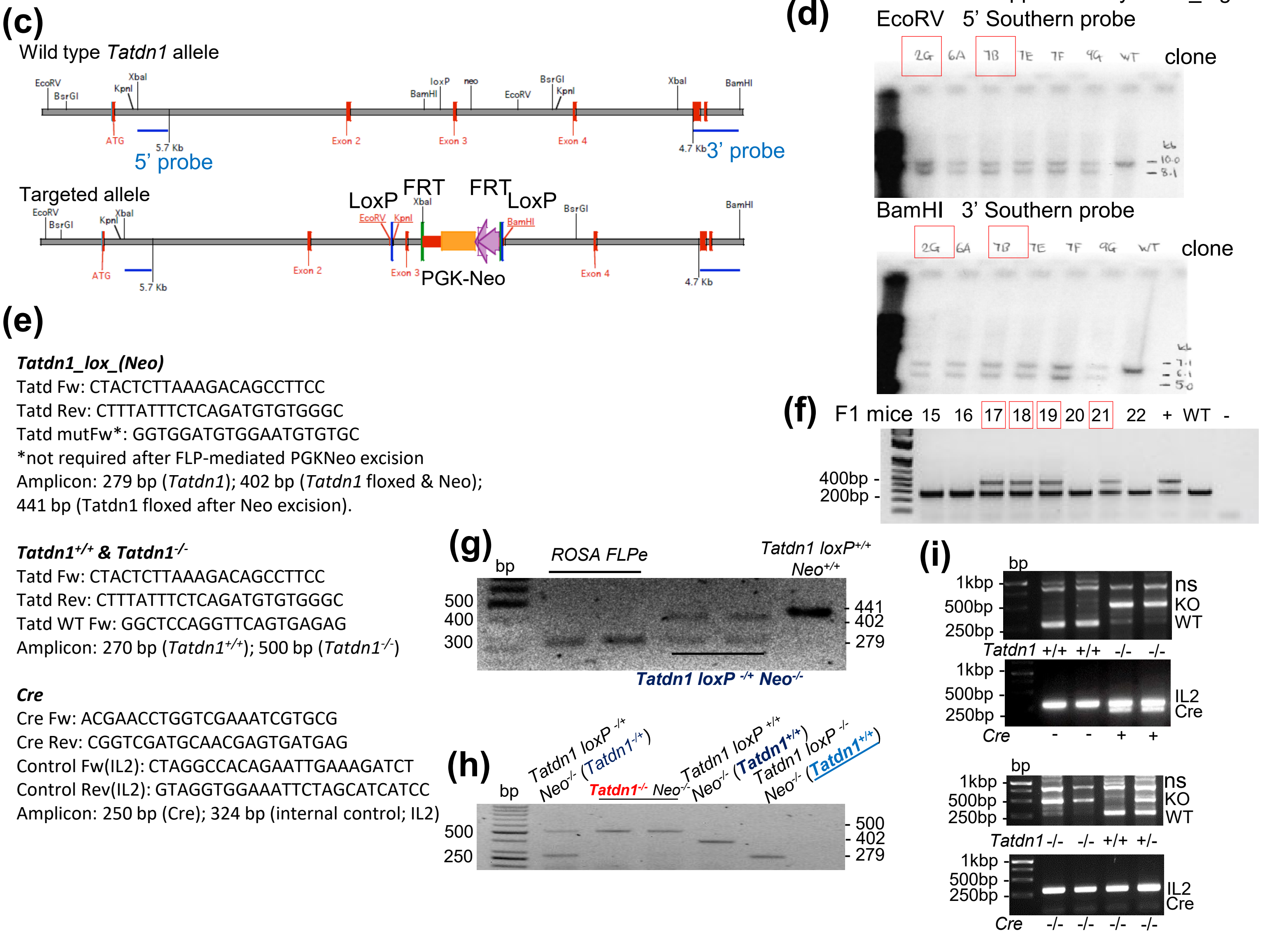

Supplementary Figure 5. Process for the conditional *Tatdn1* allele deletion in mice. (a) Fragment of the Mus musculus *Tatdn1* transcript highlighting the exonic structure and the corresponding peptide sequence below in wild type and after exon 3 (yellow) deletion in the knockout transcript, which incorporates an ORF frame shift and a stop codon in exon 4. (b) Flowchart of the procedure to obtain the $Tatd^{loxP/loxP}$ mouse described in the Materials and Methods section. (c) Scheme of the wild type and targeted alleles indicating the position of the EcoRV and BamHI restriction sites and the 5' and 3' Southern blot probes used in the ES scanning procedure. (d) Southern blot analysis of ES cell clones with the 5' probe (upper panel) and 3' probe (lower panel). (e) Genotyping primers and PCR conditions. (f) F1 genotyping, the band on top indicates the presence of the targeted allele. (g) Assessment of *NeoR* deletion after crossing with ROSA FLPe mice. (h) Assessment of exon 3 deletion after crossing with Cre mice. (i) Assessment of Cre elimination and generation of the experimental subjects (ns: not specific; IL2: internal control).

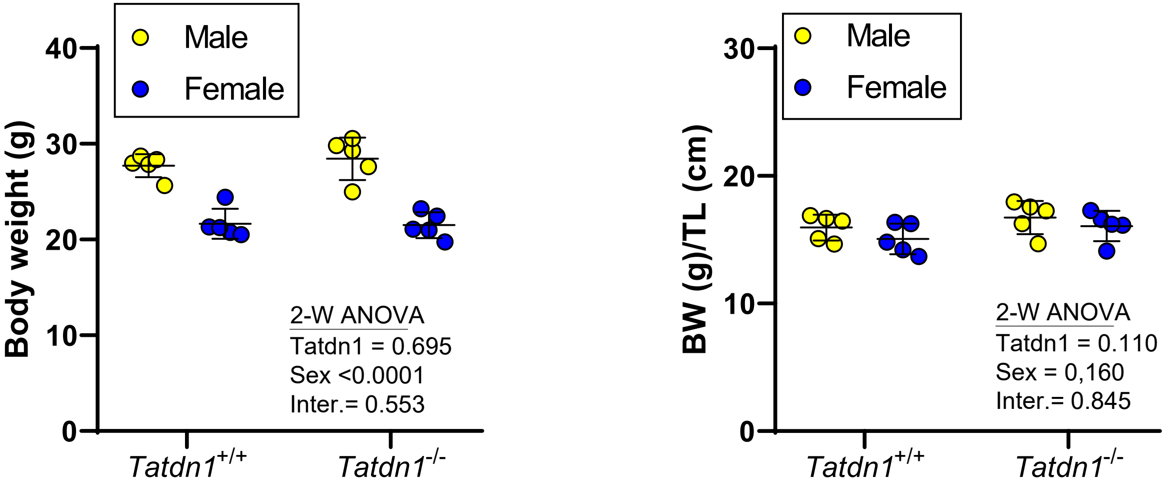

Supplementary Figure 6. Body weight of adult *Tatdn1*$^{+/+}$ and *Tatdn1*$^{-/-}$ mice, and correction by tibial length. Body weight (BW) and tibia length (TL) were measured in a set of 5 seventy-day-old males and 5 females per genotype. Two-way ANOVA was performed with BW and BW/TL data. P values are shown for either variable and their interaction.

Supplementary Figure 7. Summary of the most relevant published knowledge about TatD/TATDN1

	Wexler et al., JBC 2000 doi: 10.1074/jbc.M000800200.	Parrish & Xue, Mol Cell 2003 doi: 10.1016/s1097-2765(03)00095-9	Qiu et al., JBC 2005 DOI 10.1074/jbc.M413547200	BoseDasgupta et al., CDD 2008 doi:10.1038/cdd.2008.85	Matos et al., EMBO Rep 2009 doi:10.1038/embor.2009.34	Gannavaram et al., Mol Microb 2012 doi:10.1111/j.1365-2958.2012.07978.x	Yang et al., Cell Cycle 2012 DOI: 10.4161/cc.22886	Chen et al., Nuc Acid Res 2014 doi: 10.1093/nar/gku732	Chang et al., Nat Commun 2016 DOI: 10.1038/ncomms11537	Dorival et al., Nuc Acid Res 2023 doi: 10.1093/nar/gkad133	**Barés et al.**
Organism	**Bacteria**	C. elegans	Yeast	Leishmania	**Bacteria**	Trypanosoma	Zebra fish	**Bacteria**	Plasmodium	Human recombinant	**Mouse Rat**
Sample origin	Recomb. EcTatD (1µg)	–	Recomb. ScTatD (0-1µM)	Apoptotic nuclei	endogenous	Recomb. TbTatD produced in E coli	Recomb. DrTatDN1 (1µg)	Recomb. EcTatD (0.5-1µmol/L)	Recomb. PfTatD-GST (1-30µmol/L)	Recomb. TATDN1 (10-20µg)	**endogenous**
Conditions	In vitro	–	In vitro	In vitro	In cell	In vitro In cell (overexpr.)	In vitro	In vitro	In vitro	In vitro	**In cell In vitro**
Substrate	0.1µg linear pBS 1h 37º	–	0.4µg linear pET28b 15' 30º ^{32}P-Oligonuc.	Chromatin from apoptotic L. nuclei	Overexpression of mutated protein substrate	1µg linear plasmid 30' 30º (in vitro) TUNEL (in vivo)	0,2µg coiled pUC19 30' 37º	40nmol/L ^{32}P-Oligonuc. SsDNA&RNA 1h37º	20ng nude ds-HsDNA 30'37º	10-100nM 25mers ds, ssDNA Abasic DNA	Genomic DNA in cell (necrosis/apoptosis) In vitro 1µg linear pcDNA3 1h 37º
Ions	**5mM $MgCl_2$** (required)	–	**5mM $MgCl_2$**	**5.2mM $MgCl_2$**	–	**2-5mM $MgCl_2$**	**20mM $MgCl_2$**	**2mM $MgCl_2$**/Mn No active Ca^{2+}	**Mg^{2+} (inhibits)**	**2-10mM $MgCl_2$** $CaCl_2$ required for AP endoDNase	No DNase: In cell conditions In vitro ± 2mM Mg^{2+}
Nuclease function	**dsDNase**	RNAi= ↑TUNEL+	Endo/exo **dsDNase** KO= ↑TUNEL+	Not assessed alone ↓EndoG-TatD= change DNA degradation pattern	No. ΔTatD accumulates mutated protein lacking FeS cluster	**dsDNase** in vitro. RNAi=↓TUNEL+	**Cleaves** (no degrades) **dsDNA** in vitro	Exonuclease ssDNA RNA **No dsDNase**	**dsDNase**	apurinic DNA endo 3'-exo act. pref. on ssDNA	Not found. WT vs KO array suggest RNA biology/protein trafficking
Location	Cytoplasm (E coli)	–	–	–	–	Cytosol (IF & subfract.) Partly translocates to nucleus in cell death	–	–	Secreted? (not demonstrated)	–	Cytosol (IF & subfract. WB & proteomics) No translocation to nucleus in cell death (necr./apopt.)
Biological role (assessed)	–	RNAi= ↑cell nº	KO=↑survival ↑TUNEL/ H_2O_2	–	Degrading wild-type FeS pre-protein molecules that have failed to be exported	Cell death-related DNase	RNAi= abnormal DNA replication. polyploidy (eye)	ΔTatD ↓ survival / H_2O_2, etc.	Δtatd=↓survival Δtatd=↓ET	–	KO mouse develops dilated left ventricle and motor alterations
Comment	In vitro recomb. plasmid dsDNA degradation	RNAi=↑DNA degr. ↑TUNEL	KO=↑DNA degr ↑TUNEL	TatD activity not assessed alone Dnase could be EndoG	Endogenous/in cell No DNase	In vitro linearizes DNA Endogenous/in cell. No DNase	In vitro cleaves DNA DNA decatenation.	In vitro recomb. Do not degrade dsDNA KO=low survival relation to Dnase not shown	Blood [Mg] would inhibit PfTatd?	DNase activity not assessed with native DNA/chromatin. Low activity Recomb./in vitro	Role in DNA processing not ruled out in other conditions.

TUNEL= an indicator of nuclear DNA cleavage, not degradation. Ds= double strand; ss= single strand; ET= extracellular trap (contains DNA). Δ=deletion.

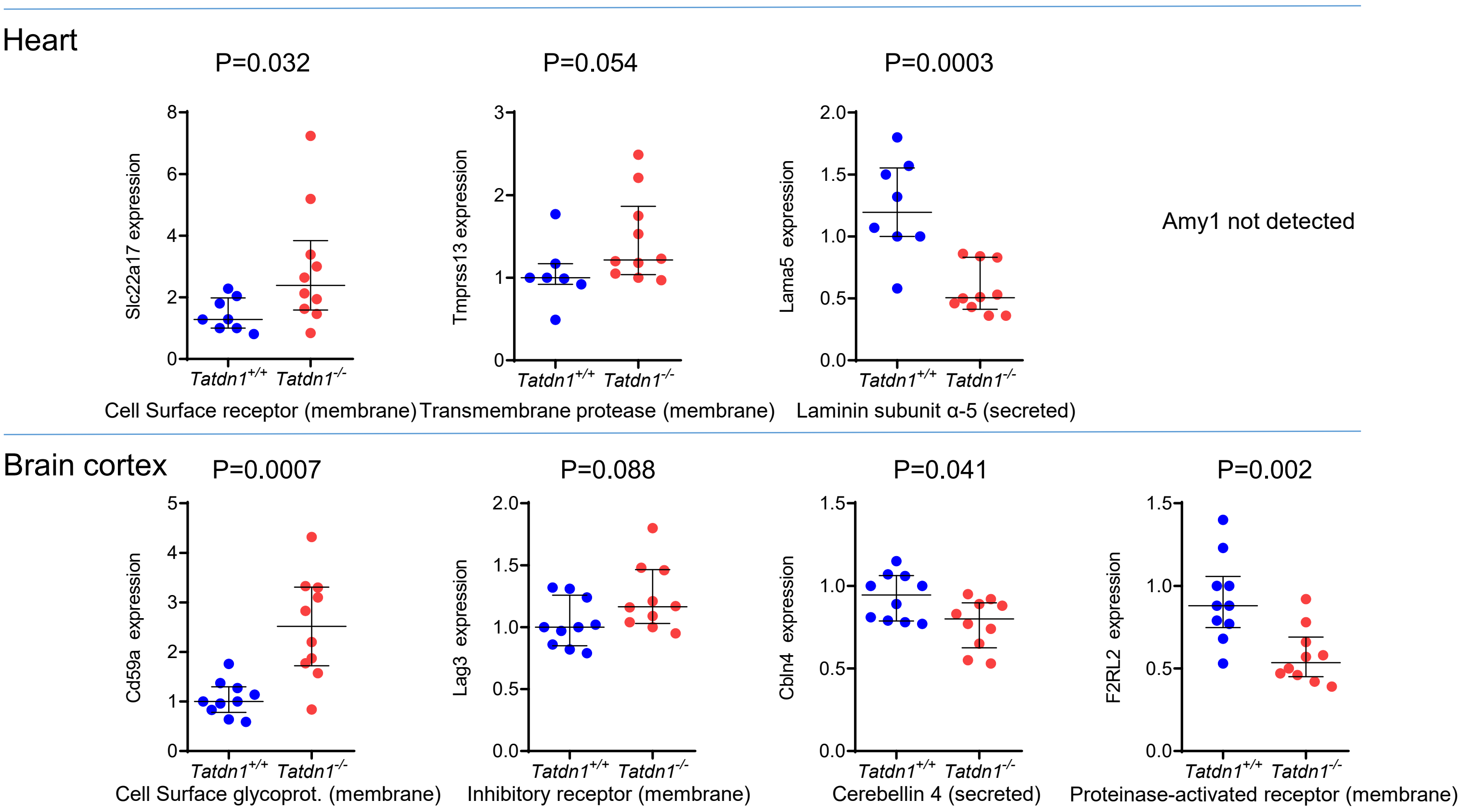

Supplementary Figure 8. Validation of microarray data from the heart and brain of *Tatdn1*$^{+/+}$ and *Tatdn1*$^{-/-}$ mice. Cardiac ventricles and brain cortex were dissected, total RNA was obtained, and reverse transcription was performed as described in the Methods section of the main text from 7-10 *Tatdn1*$^{+/+}$ and 10 *Tatdn1*$^{-/-}$ adult mice. Quantitative PCR was performed as described in the Methods section in 2 sets including *Tatdn1*$^{+/+}$ and *Tatdn1*$^{-/-}$. Data are expressed as fold vs. one of the *Tatdn1*$^{+/+}$ samples run the same day. Individual independent values (corresponding to triplicate means) are plotted plus median ± interquartile range. Mann-Whitney U test was performed, and exact P values are shown on top of each graph. Two genes upregulated and 2 downregulated were selected from the top list provided that commercial mouse TaqMan probe expanding exons was available. Genes and probes were: Slc22a17, Mm00480684_g1; Tmprss13, Mm01179270_m1; Lama5, Mm01222020_gH; Amy1, Mm01316918_m1; Cd59a, Mm01276238_m1; Lag3, Mm01185093_g1; Cbln4, Mm00558663_m1; F2rl2, Mm00438852_m1; Gapdh, Mm99999915_g1. All probes were FAM-MGB; Thermo-Fisher. Amylase-1 expression was below the detection threshold. A short description of the protein identity/function is detailed below each graph (UniProt entry).

(a)

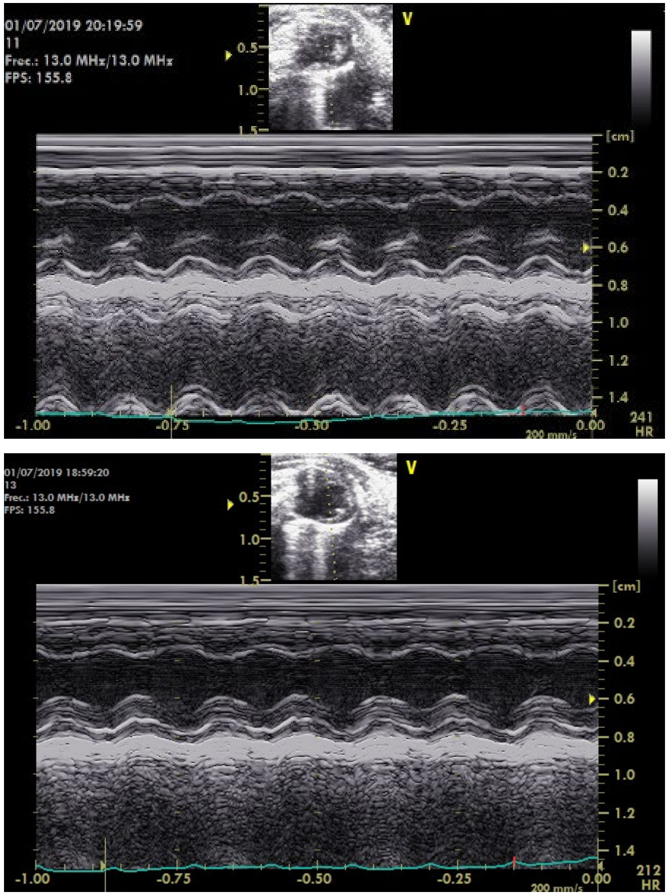

(b)

Young

	Tatdn1 $^{+/+}$	*Tatdn1* $^{-/-}$	t test	*Tatdn1* $^{+/+}$	*Tatdn1* $^{-/-}$	t test
	♂ (n=6)	♂ (n=6)	♂ *vs.* ♂	♀ (n=7)	♀ (n=6)	♀ *vs.* ♀
BW (g)	31.82 ± 1.46	31.8 ± 0.74		26.69 ± 1.94	28.25 ± 2.33	
HR (beats/min)	478.33 ± 12.2	453.83 ± 13.0		462.29 ± 17.7	467.17 ± 28.1	
LVPWT (mm)	0.68 ± 0.01	0.69 ± 0.01		0.65 ± 0.01	0.64 ± 0.02	
IVS (mm)	0.78 ± 0.01	0.77 ± 0.01		0.78 ± 0.02	0.73 ± 0.03	
LVEDD (mm)	3.88 ± 0.09	4.27 ±0.04	p=0.002	3.72 ± 0.08	4.01 ± 0.08	p=0.023
LVESD (mm)	2.47 ± 0.08	2.92 ± 0.08	p=0.003	2.52 ± 0.07	2.8 ± 0.08	p=0.021
LVEDV (μL)	65.23 ± 3.63	81.85 ± 1.75	p=0.002	59.15 ± 2.83	70.47 ± 3.16	p=0.021
LVESV (μL)	21.96 ± 1.76	32.97 ± 2.08	p=0.002	23.01 ± 1.59	29.78 ± 1.94	p=0.019
FS (%)	33.13 ± 0.83	31.67 ± 1.59		32.17 ± 1.26	30.18 ± 0.91	
EF (%)	68.63 ± 1.10	66.14 ± 2.12		66.99 ± 1.73	64.01 ± 1.78	

Old

	Tatdn1 $^{+/+}$	*Tatdn1* $^{-/-}$	t test	*Tatdn1* $^{+/+}$	*Tatdn1* $^{-/-}$	t test
	♂ (n=10)	♂ (n=6)	♂ vs. ♂	♀ (n=4)	♀ (n=4)	♀ vs. ♀
BW (gr)	39.7 ± 1.14	42.4 ± 2.84		31.0 ± 1.17	28.5 ± 1.61	
HR (beats/min)	440.4 ± 7.14	437.33 ± 6.69		454.75 ± 19.02	445.25 ± 23.12	
LVPWT (mm)	0.68 ± 0.01	0.68 ± 0.02		0.65 ± 0.02	0.63 ± 0.01	
IVS (mm)	0.82 ± 0.01	0.80 ± 0.01		0.75 ± 0.02	0.72 ± 0.02	
LVEDD (mm)	4.12 ± 0.10	4.26 ± 0.05		3.84 ± 0.09	4.14 ± 0.03	p=0.020
LVESD (mm)	2.90 ± 0.09	3.02 ± 0.04		2.74 ± 0.07	2.98 ± 0.09	
LVEDV (μL)	75.59 ± 4.03	81.31 ± 2.38		63.58 ± 3.52	75.90 ± 1.45	p=0.017
LVESV (μL)	32.54 ± 2.30	35.65 ± 1.12		28.11 ± 1.85	34.48 ± 2.74	
EF (%)	63.73 ± 1.12	62.51 ± 0.74		61.00 ± 1.31	60.84 ± 3.23	
FS (%)	29.76 ± 0.75	29.12 ± 0.53		27.92 ± 0.85	28.11 ± 1.97	

Supplementary Figure 9. Echocardiographic data complementing Fig.6. (a) Transthoracic echocardiography images corresponding to Figure 6a indicating the position of the probe. (b) **s**ummary of echocardiographic data from *Tatdn1* $^{+/+}$ (6♂ and 7♀) and *Tatdn1* $^{-/-}$ ((6♂ and 6♀) young mice, and *Tatdn1* $^{+/+}$ (10♂ and 4♀) and *Tatdn1* $^{-/-}$ ((6♂ and 4♀) old mice, complementary to Figure 6b. BW: body weight, HR: Heart rate, LVPWT: Left ventricle posterior wall thickness, IVS: interventricular septum, LVEDD: Left ventricle end diastolic diameter, LVESD: Left ventricle end systolic diameter, LVEDV: Left ventricle end diastolic volume, LVESV: Left ventricle end systolic volume, FS: Fractional shortening, EF: Ejection fraction. Data are mean ± SEM. Individual values are shown in the Source Data file. Statistics: P values after Unpaired t-test comparing same sex of both genotypes are shown.

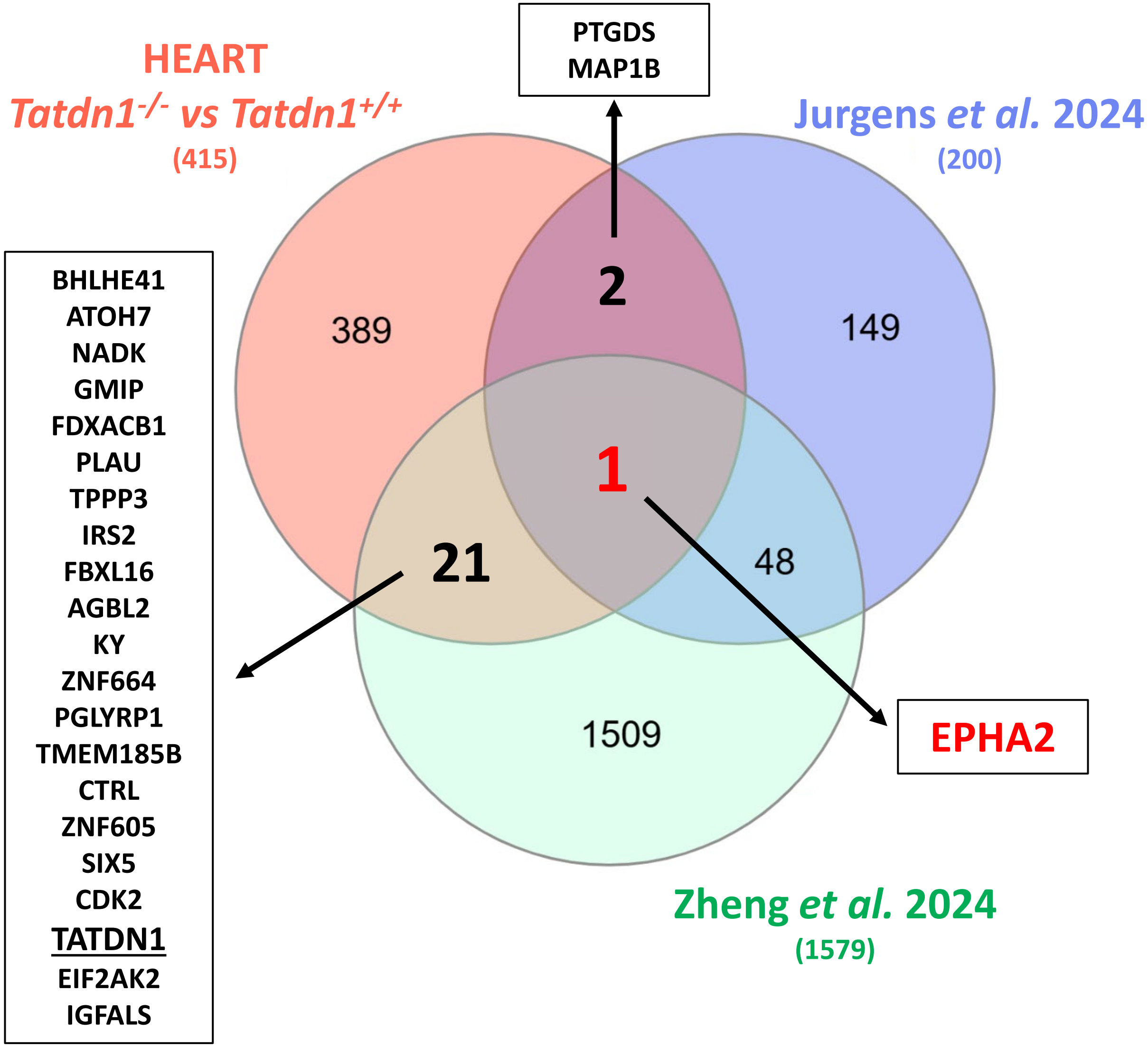

Supplementary Figure 10. Venn diagram illustrating differentially expressed genes (DEGs) shared across datasets of cardiac genes differentially expressed in *Tatdn1$^{-/-}$* vs. *Tatdn1$^{+/+}$* mice (this article), Jurgens et al. 2024 and Zheng et al., 2024. The red circle represents DEGs in the heart of *Tatdn1$^{-/-}$ vs. Tatdn1$^{+/+}$* mice ($log_2FC > 0.5$ or $log_2FC < -0.5$, $p < 0.05$), the blue circle represents DEGs from Jurgens, S.J. et al. Nat Genet 2024, and the green circle represents DEGs from Zheng, S.L et al. Nat Genet 2024. Numbers within each section indicate the count of unique or shared DEGs. *EPHA2* (highlighted) is the only gene common to all three datasets. The diagram was generated using Interactivenn.

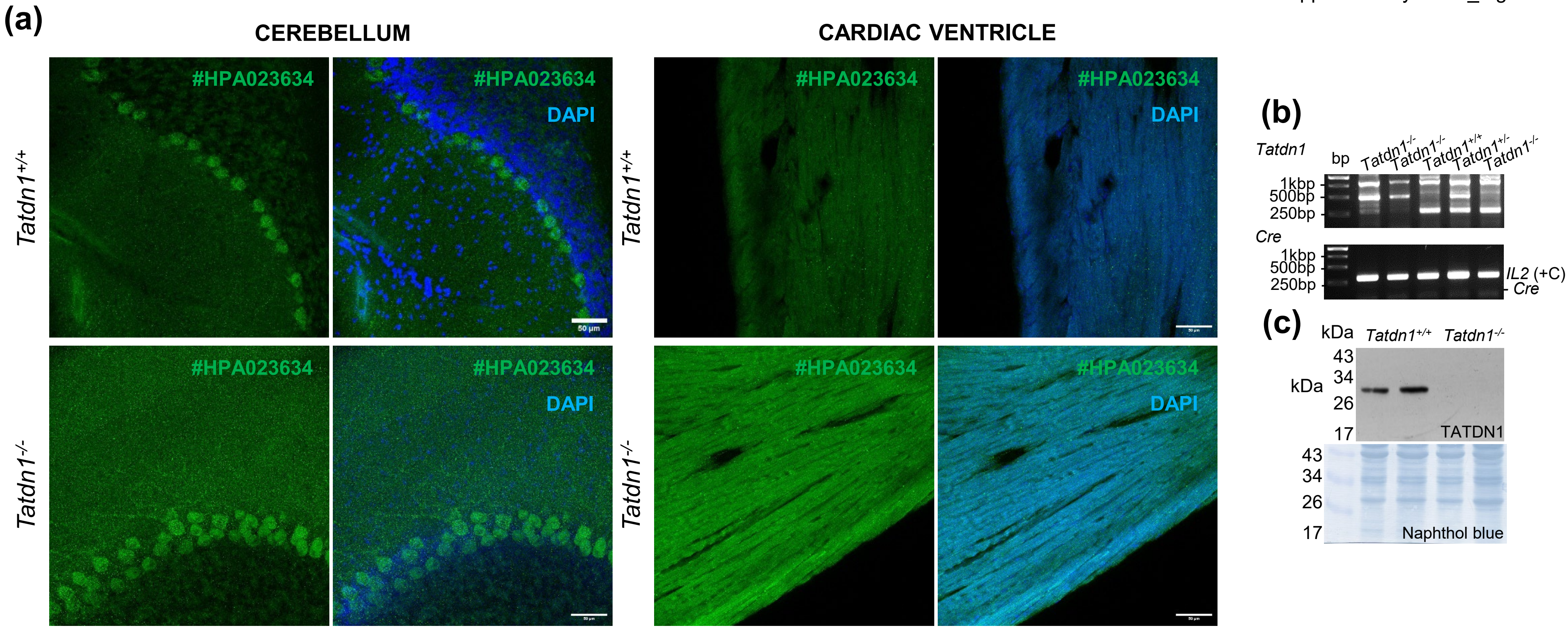

Supplementary Figure 11. (a) Immunofluorescence analysis of paraformaldehyde fixed cerebellar and cardiac tissues of *Tatdn1*$^{+/+}$ and *Tatdn1*$^{-/-}$ adult mice using TATDN1 antibody (SIGMA #HPA023634; Human Protein Atlas: https://www.proteinatlas.org/ENSG00000147687-TATDN1/summary/antibody). Secondary Cy3- coupled antibody (1:200; Jackson ImmunoResearch Laboratories, #715-165-150) was used for visualization. Nuclei were stained with 4′,6-diamidino-2- phenylindole (DAPI; catalogue #D9542, Sigma-Aldrich). Scale bar: 50µm. (b) Tatdn1 and Cre genotyping of the litter (see Suppl. Fig. 4i for information), IL2, PCR positive control. (c) TATDN1 detection by Western blot using the same batch of #HPA023634 TATDN1 antibody in total protein extracts of cardiac ventricles from littermates.

Methods: *Immunofluorescence detection of TATDN1 in histological sections*

Animals were deeply anaesthetized and subsequently intracardially perfused with 4% (weight/volume) paraformaldehyde in 0.1M phosphate buffer. Brains and hearts were dissected out and kept 48h in 4% paraformaldehyde 0.1M phosphate buffer in agitation. After fixation, free-floating sagittal sections of brains (30 µm) or hearts (70 µm) were obtained using a vibratome (Leica, VT1000). Sections were first washed in PBS and incubated in 50mM NH_4Cl, for 30 minutes. Blocking and permeabilization were performed for 1h in PBS-T with 0.02% azide, 3% NGS and 0.2% BSA. Primary antibody (TATDN1, 1:100, #HPA023634, Sigma) was diluted in blocking solution and sections were incubated overnight at 4ºC in agitation. Secondary Cy3- coupled antibody (1:200; Jackson ImmunoResearch Laboratories, #715-165-150) was diluted in blocking solution and tissues were incubated for 2h at room temperature. Nuclei were stained for 10 min with 4′,6-diamidino-2- phenylindole (DAPI; catalogue #D9542, Sigma-Aldrich). The sections were mounted onto slides and cover-slipped with Mowiol. Images were taken with a Leica Confocal SP5 with a 40x oil-immersion objective. The pixel resolution used was 1024x1024 and the z-step size was 4 µm.
